# Executive Dysfunction and Memory Impairments in Hikikomori: A Neuropsychological Case-Control Study from Oman

**DOI:** 10.12688/f1000research.181828.2

**Published:** 2026-06-23

**Authors:** Nutaila Al Kharusi, Yahya M. Al-Farsi, Moon Fai Chan, Samir Al-Adawi, Mallak Al-Mandhari, Nasser Al-Sibani

**Affiliations:** 1 Department of Family Medicine & Public Health, Sultan Qaboos University College of Medicine and Health Science, Muscat, Muscat Governorate, 123, Oman; 2 Behavioral Medicine, Sultan Qaboos University College of Medicine and Health Science, Muscat, Muscat Governorate, 123, Oman

**Keywords:** Hikikomori, Social Withdrawal, Case-Control Study, Neurocognition, CANTAB, Executive Function, Memory Recall, Oman.

## Abstract

**Background:**

Hikikomori, a form of prolonged social withdrawal, has been widely studied in East Asia but remains underexplored in the Middle East. Cultural and social factors may influence its cognitive profile, necessitating localized research.

**Aim:**

To compare cognitive performance between Hikikomori cases and healthy controls in Oman using the Cambridge Neuropsychological Test Automated Battery (CANTAB), focusing on executive function, memory recall, and processing speed.

**Methods:**

A case-control study was conducted in 2024 with 150 participants (54 Hikikomori cases, 96 controls) recruited from a tertiary hospital in Oman. Hikikomori cases were classified using the HiDE-I structured diagnostic interview, applying a broad definition that included pathological, at-risk, and resembling Hikikomori categories. CANTAB was used to assess key cognitive domains. Independent t-tests and linear regression analyses were performed to compare outcomes.

**Results:**

Hikikomori cases showed significant impairments in visual processing accuracy (p < 0.001), response latency (p = 0.004), immediate recall (p = 0.031), delayed recall (p = 0.009), and planning efficiency (p < 0.001) compared to controls. Adjusted regression analyses—controlling for age, gender, education, income, employment, marital status, and governorate—confirmed that Hikikomori status was significantly associated with lower visual processing accuracy (β = −0.453, p = 0.001), slower response latency (β = 0.266, p = 0.001), poorer immediate (β = −0.201, p = 0.019) and delayed recall (β = −0.255, p = 0.003), and reduced planning efficiency (β = −0.453, p = 0.001). No significant differences were found for spatial working memory tasks.

**Conclusion:**

Hikikomori individuals in Oman exhibit significant deficits in executive function, memory recall, processing speed, and visual attention, even after adjusting for socio-demographic factors. These findings underscore the importance of early neurocognitive screening and tailored interventions. This is one of the first studies to explore Hikikomori’s cognitive profile in the Middle East.

**List of abbreviations**
B/β: Beta Coefficient
CANTAB: Cambridge Neuropsychological Test Automated Battery
CI: Confidence Interval
HiDE-I : Hikikomori Diagnostic Evaluation Interview
HLID: Hikikomori-like Idiom of Distress
IED: Intra/Extra Dimensional Set Shift
OTSMDLFC: One Touch Stockings of Cambridge – Mean Latency to First Choice
OTSPSFC: One Touch Stockings of Cambridge – Problems Solved on First Choice
PRMPCI: Pattern Recognition Memory – Percent Correct Immediate
PRMPCD: Pattern Recognition Memory – Percent Correct Delayed
RVPA: Rapid Visual Processing Accuracy
RVPMDL: Rapid Visual Processing Mean Response Latency
SAD: Social Anxiety Disorder
SQUH: Sultan Qaboos University Hospital
SSP: Spatial Span
SWMBE4: Spatial Working Memory – Between Errors (4 Boxes)
SWMBE6: Spatial Working Memory – Between Errors (6 Boxes)
SWMBE8: Spatial Working Memory – Between Errors (8 Boxes)
SWMBE468: Spatial Working Memory – Between Errors (4, 6, 8 Boxes Combined)
SWMS: Spatial Working Memory – Strategy Score
SD: Standard Deviation
SE: Standard Error
SPSS: Statistical Package for the Social Sciences

## Introduction

Hikikomori, a severe form of social withdrawal where individuals isolate themselves for extended periods avoiding school, work, and social interactions, was initially documented in Japan and has since been recognized globally, including in the Middle East ( Kato et al., 2019; Kato et al., 2020, 2024; Saito, 1998; Tan et al., 2021; Teo et al., 2023). This condition is often comorbid with psychiatric disorders such as social anxiety disorder (SAD) and depression, which are characterized by cognitive impairments—such as deficits in executive function, attentional control, and working memory—further reinforcing social withdrawal ( Airaksinen et al., 2005; Bourke et al., 2012; Kim et al., 2014; Luciana, 2003; Rhodes et al., 2005; Teo et al., 2020).

In Oman, a high-income Arab country with rapid social changes and evolving family structures, Hikikomori-like behaviors are emerging amid increasing academic and career pressures ( Al-Sibani et al., 2023; Sakamoto et al., 2005). Despite a population of approximately 5.1 million as of 2023 ( World Bank, 2023), mental health services are underutilized due to stigma and limited awareness, potentially obscuring the true prevalence of Hikikomori in the region ( Al Alawi et al., 2017; Albadi et al., 2025). Recent findings in Oman indicate significant subgroups exhibiting Hikikomori-like social withdrawal ( Al-Sibani et al., 2023; Chan et al., 2024). While earlier work operationalized this presentation as Hikikomori-like idiom of distress (HLID), the present study focuses specifically on clinically classified Hikikomori cases defined using the HiDE-I. Additionally, the increased sensitivity to social evaluation, a hallmark of SAD, reinforces avoidant behaviors in individuals with Hikikomori ( Sakamoto et al., 2005).

Research shows that social withdrawal in Hikikomori is strongly associated with psychiatric comorbidities such as depression, anxiety, and avoidant personality disorder ( Bowker et al., 2019; Cai et al., 2023; Chauliac et al., 2017; Pozza et al., 2019; Teo et al., 2020). A survey found that 54.5% of those meeting Hikikomori criteria had a psychiatric disorder, with anxiety and depression being the most common ( Kato et al., 2024).

From a neurocognitive perspective, we hypothesized that individuals with Hikikomori would demonstrate impairments primarily in executive and attentional domains—particularly planning efficiency, processing speed, and memory recall—rather than in basic spatial working memory. These domains are consistently implicated in depression, anxiety, and avoidant psychopathology, which frequently co-occur with Hikikomori. In contrast, spatial working memory deficits were not expected to be a core feature of Hikikomori itself but may emerge secondarily in more severe or clinically complex presentations.

Given Oman’s collectivist culture and rapid modernization, Hikikomori-like social withdrawal is emerging as a culture-reactive phenomenon rather than one unique to Japan ( Al-Sibani et al., 2023). Al-Sibani et al. (2023) reported that 44% of participants exhibited HLID following the COVID-19 pandemic, with risk factors such as screen overuse, unemployment, mental illness, and adverse childhood experiences, echoing global Hikikomori patterns. These findings align with regional data from the United Arab Emirates, where problematic social media use, gaming behavior, and loneliness were significantly associated with Hikikomori-like traits, suggesting digital over-engagement as a potential pathway into social withdrawal ( Shah et al., 2024).

To explore cognitive impairments associated with this condition, this study employed the Cambridge Neuropsychological Test Automated Battery (CANTAB)—a non-verbal, examiner-independent tool that minimizes cultural and language biases ( Chamberlain et al., 2011; J. Fray et al., 1996; Luciana, 2003; Mehta et al., 2004; Rhodes et al., 2005). CANTAB assesses core domains such as executive function, memory, attention, and response inhibition, and has been validated in both clinical and non-clinical populations across diverse cultural contexts ( Airaksinen et al., 2005; Bourke et al., 2012; Green et al., 2019; Kim et al., 2014).

This study aims to compare the cognitive performance of Hikikomori cases and healthy controls in Oman, using neuropsychological assessments to identify deficits in executive function, memory recall, and processing speed. Given the lack of research on Hikikomori in the Middle East, this study also seeks to examine socio-demographic characteristics of Hikikomori cases versus non-cases among attendees of the Behavioral Medicine Clinic in Oman. By comparing cognitive performance using CANTAB, this research will provide insights into potential neurocognitive risk factors associated with Hikikomori and contribute to the understanding of social withdrawal syndromes within the Omani context.

To our knowledge, this is the first study to examine Hikikomori individuals using CANTAB in the Middle East. This study builds on prior work evaluating the diagnostic performance of the HQ-25 against the clinician-administered HiDE-I in Oman and uses HiDE-I–based case classification to examine whether individuals with Hikikomori also show measurable neurocognitive differences. This allows the analysis to move beyond screening-level identification and focus on cognitively relevant differences among clinically classified cases.

## Methods

### Study design and setting

This case–control study was conducted at the Behavioral Medicine Department of Sultan Qaboos University Hospital (SQUH), a tertiary care teaching hospital in Muscat, Oman. The study was nested within a larger cross-sectional investigation that aimed to identify Hikikomori-like idiom of distress (HLID) among adults attending the department. Initial screening was carried out using the Hikikomori Questionnaire-25 (HQ-25), alongside the collection of socio-demographic information. Participants were subsequently invited to complete a structured diagnostic interview using the HiDE-I to allow for accurate case classification. Following diagnostic assessment, a subset of participants was selected by convenience sampling to undergo neuropsychological testing using the Cambridge Neuropsychological Test Automated Battery (CANTAB), which assessed executive function, working memory, attention, and response inhibition. Hikikomori case status in the present study was determined using the clinician-administered HiDE-I rather than HQ-25 screening scores alone.

### Participants

The initial cross-sectional sample consisted of 1,200 Omani nationals aged between 18 and 59 years, recruited from outpatients attending the Behavioral Medicine Department at SQUH and their accompanying attendees. Of these, 454 participants completed the HiDE-I structured diagnostic interview. From this group, 150 participants were selected to complete CANTAB testing, including 85 patients and 65 attendees. Patients were individuals attending the department for psychiatric care. Attendees were accompanying family members or companions who were not themselves seeking psychiatric treatment and had no known psychiatric diagnosis. Cognitive performance outcomes were examined across multiple domains. The importance of each cognitive domain was evaluated using standardized β coefficients derived from adjusted regression models ( Figure 1).

**Figure 1.**
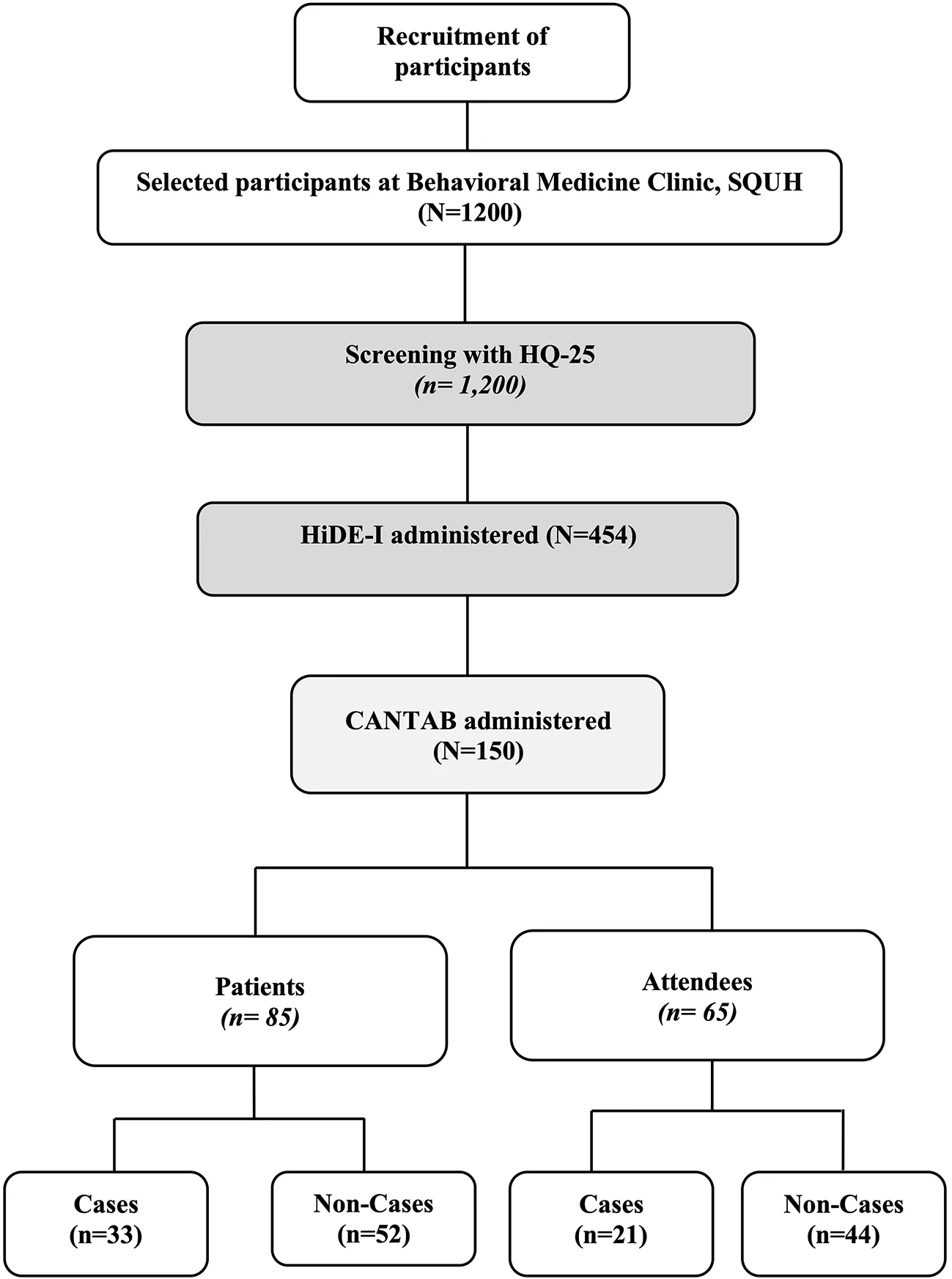
Recruitment and classification of participants at the Behavioral Medicine Clinic, SQUH, Oman, 2024.

### Case ascertainment & selection of cases

Hikikomori cases were identified using the Hikikomori Diagnostic Evaluation Interview (HiDE-I), a structured clinical assessment tool that aligns with international diagnostic criteria for prolonged social withdrawal ( Teo et al., 2023).

For the purposes of this neurocognitive case-control study, a broad HiDE-I case definition was used. This definition classified participants as Hikikomori cases if they met criteria for any HiDE-I category: pathological Hikikomori, at-risk Hikikomori, or Hikikomori resemblance. Participants who did not meet criteria for any of these three categories were classified as non-cases. This broader definition was deliberately adopted because cognitive differences associated with social withdrawal may not be limited to fully pathological Hikikomori but may also be present in earlier or subthreshold stages of withdrawal.

Pathological Hikikomori cases were those who had been socially withdrawn for six months or more, with significant psychological distress or functional impairment. At-risk Hikikomori referred to individuals showing early signs of social withdrawal but not meeting full diagnostic criteria, while Hikikomori resemblance applied to those exhibiting some withdrawal behaviors but lacking clear impairment.

A total of 54 participants were identified as Hikikomori cases and agreed to participate in the study, yielding a response rate of 93%. All cases were evaluated by two trained mental health professionals, each experienced in assessing social withdrawal disorders. Inter-rater reliability was assessed using percent agreement between two independent clinicians who conducted the HiDE-I interviews. Initial agreement on Hikikomori classification across the three subtypes was 96%. Discrepant cases were subsequently reviewed through a structured consensus adjudication process involving joint discussion of interview data and clinical criteria, after which full agreement (100%) was reached. This consensus-based approach was used to ensure diagnostic accuracy rather than to inflate reliability estimates.

### Selection of controls

Control participants were recruited from the Behavioral Medicine Department at SQUH and confirmed to be Hikikomori-free through the Hikikomori Diagnostic Evaluation Interview (HiDE-I). To be included, participants were required to have no history of prolonged social withdrawal and not meet criteria for any HiDE-I subcategory, including pathological, at-risk, or resemblance Hikikomori. A total of 96 control participants consented to participate, yielding a response rate of 87%.

Inter-rater reliability for control classification was assessed using percent agreement between two independent clinicians who conducted the HiDE-I interviews. Initial agreement on control status was 95%. Discrepant cases were subsequently reviewed through a structured consensus adjudication process involving joint discussion of interview data and clinical criteria, after which full agreement (100%) was reached. This consensus approach was applied to ensure accurate exclusion of Hikikomori rather than to inflate reliability estimates.

### Study tools

Sociodemographic data were collected using a structured questionnaire. In addition, participants underwent cognitive testing using the Cambridge Neuropsychological Test Automated Battery (CANTAB) ( J. Fray et al., 1996). CANTAB is a computerized neuropsychological assessment tool widely used in psychiatric research to measure executive function, attention, working memory, and processing speed. Testing was conducted via touchscreen devices, with responses recorded automatically. A trained examiner provided standardized instructions to ensure comprehension. The testing was conducted in a quiet environment, and headphones were provided when necessary to minimize auditory distractions.

CANTAB assessed multiple cognitive domains relevant to this study. Executive function was measured using the Intra/Extra Dimensional Set Shift (IED), which evaluates cognitive flexibility, and the One Touch Stockings of Cambridge (OTS), which assesses planning and problem-solving skills. Working memory was tested using the Spatial Working Memory (SWM) and Spatial Span (SSP) tasks. Attention and processing speed were assessed through the Rapid Visual Information Processing (RVP) task, while Pattern Recognition Memory (PRM) was used to evaluate visual memory recall. The CANTAB tasks and corresponding outcome measures used in this study are summarized in Table 1.

**Table 1.** CANTAB Tasks and outcome measures used in the cognitive assessment.

Cognitive domain	CANTAB task	Outcome variable (used in analyses)	Description
Visual attention & processing speed	Rapid Visual Information Processing (RVP)	RVPA RVPMDL	Proportion of correct detections (visual processing accuracy) Mean response latency (processing speed)
Visual memory	Pattern Recognition Memory (PRM)	PRMPCI PRMPCD	Percentage correct on immediate recall Percentage correct on delayed recall
Executive function (planning)	One Touch Stockings of Cambridge (OTS)	OTSPSFC OTSMDLFC	Number of problems solved on first choice (planning efficiency) Mean latency to first choice (planning latency)
Working memory	Spatial Working Memory (SWM)	SWMBE468 SWMBE4 SWMBE6 SWMBE8	Total between-search errors (all box sizes) Errors at 4-box stage Errors at 6-box stage Errors at 8-box stage
Strategy use	Spatial Working Memory (SWM)	SWMS	Strategy score (lower scores indicate more efficient strategy use)

### Data collection

Data was collected through standardized interviews and computerized cognitive assessments. Trained mental health professionals administered all assessments following standardized protocols. HiDE-I interviews took place in private clinical settings, and CANTAB assessments were conducted in a controlled environment with sufficient time for task completion. Assessors administering the CANTAB were blinded to participants’ Hikikomori case or control status to reduce the risk of performance and observer bias.

### Statistical analysis

Data were analyzed using SPSS version 27.0 (IBM Corp., Armonk, NY, USA). Descriptive statistics were used to summarize participant characteristics, with means and standard deviations reported for continuous variables and frequencies with percentages for categorical variables. Independent samples t-tests were conducted to compare cognitive performance between Hikikomori cases and controls. Associations between Hikikomori status and cognitive performance were examined using linear regression models, including both crude models and adjusted models controlling for age, gender, education level, income, employment status, governorate, and marital status. Regression assumptions were assessed prior to analysis. Normality of residuals was evaluated using visual inspection of histograms and Q-Q plots, while homoscedasticity and linearity were assessed through residual scatterplots. Reaction time and latency measures, which showed mild positive skew, were retained in their original scale, as residual diagnostics indicated acceptable approximation to normality and model fit. No variable transformations were required. To ensure robustness of findings, non-parametric sensitivity analyses were also performed, including Mann–Whitney U tests for group comparisons and Spearman’s rank correlation coefficients for associations. Results from these supplementary analyses were consistent with the parametric findings.

Sample size was calculated prior to data collection using Rothman’s Episheet for case-control power estimation, with a control-to-case ratio of 1:1, Z-alpha of 1.96, and an exposure prevalence of 0.15. The power curves indicated that approximately 400 total participants would be required to achieve 80% power for detecting an odds ratio of 2.0. The achieved sample of 54 cases and 96 controls (N = 150 total) provided approximately 66% power to detect an OR of 2.0 and was adequately powered to detect larger effect sizes (OR ≥ 3.0, power > 0.98). The study was therefore sufficiently powered for the primary between-group comparisons, which yielded several significant findings with medium-to-large effect sizes. However, the sample was underpowered to detect smaller effects, and non-significant findings—particularly for spatial working memory domains—should be interpreted as inconclusive rather than as evidence of absence.

## Results

### Socio-demographic characteristics Hikikomori cases and non-cases 

Table 2 presents the socio-demographic characteristics of participants who underwent CANTAB testing. Of the 150 participants included, 54 were classified as Hikikomori cases and 96 as non-cases based on the HiDE-I broad definition. Patients constituted 56.7% of the sample (n = 85), while attendees accounted for 43.3% (n = 65).

**Table 2.** Socio-demographic characteristics of Hikikomori cases and non-cases undergoing CANTAB, Oman, 2024.

Characteristic	All participants (N = 150)		Patients (N = 85)		Attendees (N = 65)	
	Cases (N/%) 54	Non-cases (N/%) 96	Cases (N/%) 33	Non-cases (N/%) 52	Cases (N/%) 21	Non-cases (N/%) 44
**Gender**						
**Female**	38 (70.4)	60 (62.5)	25 (29.4)	37 (43.5)	13 (20.0)	23 (35.4)
Male	16 (29.6)	36 (37.5)	8 (9.4)	15 (17.6)	8 (12.3)	21 (32.3)
**Age categories**						
**18–29**	42 (77.8)	65 (67.7)	26 (30.6)	37 (43.5)	16 (24.6)	28 (43.1)
30–39	8 (14.8)	17 (17.7)	4 (4.7)	9 (10.6)	4 (6.2)	8 (12.3)
40 or more	4 (7.4)	14 (14.6)	3 (3.5)	6 (7.1)	1 (1.5)	8 (12.3)
**Family income**						
**<1000**	25 (46.3)	43 (44.8)	13 (15.3)	23 (27.1)	12 (18.5)	20 (30.8)
**1000–1999**	20 (37.0)	30 (31.2)	12 (14.1)	14 (16.5)	8 (12.3)	16 (24.6)
2000–2999	6 (11.1)	14 (14.6)	6 (7.1)	9 (10.6)	0 (0.0)	5 (7.7)
3000 or more	3 (5.6)	9 (9.4)	2 (2.4)	6 (7.1)	1 (1.5)	3 (4.6)
**Employment**						
Employed	8 (14.8)	31 (32.3)	6 (7.1)	14 (16.5)	2 (3.1)	17 (26.2)
**Unemployed**	46 (85.2)	65 (67.7)	27 (31.8)	38 (44.7)	19 (29.2)	27 (41.5)
**Marital Status**						
Never Married	39 (72.2)	70 (72.9)	23 (27.1)	42 (49.4)	16 (24.6)	28 (43.1)
Ever Married	15 (27.8)	26 (27.1)	10 (11.8)	10 (11.8)	5 (7.7)	16 (24.6)
**Have children**						
Yes	12 (22.2)	21 (21.9)	7 (21.2)	6 (11.5)	5 (23.8)	15 (34.1)
No	42 (77.8)	75 (78.1)	26 (78.8)	46 (88.5)	16 (76.2)	29 (65.9)
**Education years**						
Less than 12 years	0 (0.0)	4 (4.2)	0 (0.0)	0 (0.0)	0 (0.0)	4 (6.2)
12 years	16 (29.6)	25 (26.0)	9 (10.6)	12 (14.1)	7 (10.8)	13 (20.0)
Above 12 years	38 (70.4)	67 (69.8)	24 (28.2)	40 (47.1)	14 (21.5)	27 (41.5)
**Governorate**						
Buraimi	0 (0.0)	1 (1.0)	0 (0.0)	0 (0.0)	0 (0.0)	1 (1.5)
Dakhliya	10 (18.5)	10 (10.4)	4 (4.7)	3 (3.5)	6 (9.2)	7 (10.8)
Dhahirah	1 (1.9)	5 (5.2)	1 (1.2)	4 (4.7)	0 (0.0)	1 (1.5)
Sharqiyah	5 (9.3)	6 (6.2)	2 (2.4)	3 (3.5)	3 (4.6)	3 (4.6)
Batinah	17 (31.5)	21 (21.9)	11 (12.9)	9 (10.6)	6 (9.2)	12 (18.5)
Dhofar	1 (1.9)	0 (0.0)	1 (1.2)	0 (0.0)	0 (0.0)	0 (0.0)
Muscat	20 (37.0)	53 (55.2)	14(16.5)	33 (38.8)	6(9.2)	20 (30.8)

Values are presented as n (%). Percentages are calculated within column denominators (i.e., within cases and non-cases for all participants, patients, and attendees, respectively). Hikikomori case status was determined using the HiDE-I broad definition, including pathological, at-risk, and resemblance categories.

Among Hikikomori cases, the majority were female (70.4%) and aged 18–29 years (77.8%), compared with 62.5% and 67.7%, respectively, among non-cases. A higher proportion of cases were unemployed (85.2%) compared to non-cases (67.7%). Differences were also observed across family income categories, with cases more frequently represented in lower income brackets.

No substantial differences were observed between cases and non-cases with respect to marital status, parental status, educational attainment, or governorate of residence. Distributions across governorates reflected the clinic-based sample, with Muscat and Al Batinah accounting for the largest proportions in both groups.

### Cognitive performance comparison between Hikikomori cases and non-cases 

Table 3 presents comparisons of cognitive performance between Hikikomori cases and non-cases. Across the full sample, Hikikomori cases demonstrated significantly poorer performance in visual processing accuracy, response latency, immediate recall, delayed recall, and planning efficiency (p < 0.05). No significant group differences were observed for planning latency, spatial working memory errors, or strategy use.

**Table 3.** Comparison of cognitive performance metrics between Hikikomori cases and non-cases, Oman, 2024.

Domain	All participants (N = 150)			Patients (N = 85)			Attendees (N = 65)		
	Cases	Non-cases		Cases	Non-cases		Cases	Non-cases	
	M (SD)	M (SD)	P	M (SD)	M (SD)	P	M (SD)	M (SD)	P
Visual Processing Accuracy (RVPA)	0.78 (0.09)	0.85 (0.06)	0.01	0.80 (0.07)	0.86 (0.05)	0.001	0.74 (0.10)	0.84 (0.06)	0.01
Response Latency (RVPMDL)	638.48 (211.61)	539.76 (161.62)	0.01	619.39 (192.13)	535.00 (151.60)	0.04	668.48 (240.93)	544.36 (174.40)	0.04
Immediate Recall (PRMPCI)	74.69 (18.71)	81.19 (6.86)	0.03	72.73 (19.06)	82.59 (16.01)	0.01	77.78 (18.17)	79.55 (17.84)	0.71
Delayed Recall (PRMPCD)	62.45 (17.30)	70.89 (19.49)	0.01	62.96 (18.20)	73.18 (20.36)	0.02	61.64 (16.19)	68.31 (18.31)	0.16
Planning Efficiency (OTSPSFC)	6.76 (3.97)	9.83 (2.77)	0.01	7.03 (4.24)	9.92 (2.69)	0.01	6.33 (3.57)	9.72 (2.88)	0.01
Planning Latency (OTSMDLFC)	11129.70 (8,628.51)	12484.20 (5,935.00)	0.25	10951.15 (8904.45)	12815.58 (6004.42)	0.25	11441.03 (8384.38)	12092.57 (5896.67)	0.71
Spatial Working Memory Errors (All) (SWMBE468)	15.02 (9.47)	12.94 (9.59)	0.20	15.00 (10.14)	12.29 (9.71)	0.22	15.05 (8.57)	13.70 (9.51)	0.59
Strategy Use (SWMS)	8.59 (2.06)	8.21 (2.25)	0.30	8.48 (2.33)	8.34 (2.21)	0.78	8.76 (1.58)	8.05 (2.30)	0.20
Spatial Memory Errors (4 Boxes) (SWMBE4)	1.09 (1.55)	0.74 (1.22)	0.15	1.00 (1.46)	0.69 (1.20)	0.29	1.24 (1.70)	0.80 (1.27)	0.30
Spatial Memory Errors (6 Boxes) (SWMBE6)	3.87 (3.34)	3.06 (3.66)	0.18	3.91 (3.35)	2.92 (3.59)	0.21	3.81 (3.40)	3.23 (3.77)	0.55
Spatial Memory Errors (8 Boxes) (SWMBE8)	10.06 (6.39)	9.14 (7.01)	0.43	10.09 (7.13)	8.67 (7.37)	0.38	10.00 (5.19)	9.68 (6.59)	0.85

In subgroup analyses, patients with Hikikomori showed significantly poorer performance across multiple domains, including visual processing accuracy, memory recall, and planning efficiency. Among attendees, significant differences were observed for visual processing accuracy and planning efficiency; however, differences in immediate and delayed recall were not statistically significant. Supplementary nonparametric analyses (Mann–Whitney U tests) produced comparable results, supporting the robustness of these findings.

### Association between hikikomori status and cognitive performance

Table 4 presents the crude and adjusted regression models assessing the association between Hikikomori status and cognitive performance across all participants, patients, and attendees. Among all participants (N = 150), Hikikomori status was significantly associated with lower visual processing accuracy, slower response latency, poorer immediate and delayed recall, and reduced planning efficiency after adjusting for sociodemographic factors. Among patients, significant associations were found for greater spatial working memory errors, poorer memory recall, and reduced planning efficiency. Among attendees, lower visual processing accuracy and poorer memory recall remained significant. Non-significant domains included planning latency, strategy use, and spatial memory errors.

**Table 4.** Crude and adjusted linear regression models of Hikikomori status and cognitive performance, Oman, 2024.

Domain	All participants (N = 150)					Patients (N = 85)						Attendees (N = 65)						
	Crude		Adjusted			Crude			Adjusted			Crude			Adjusted			
	B	SE	P	B	SE	P	B	SE	P	B	SE	P	B	SE	P	B	SE	P
Visual Processing Accuracy (RVPA)	−0.44	0.012	0.01	−0.453	0.012	0.01	−.422	0.013	0.01	−0.408	0.014	0.01	−.506	0.021	0.001	−0.585	0.023	0.01
Response Latency (RVPMDL)	0.255	30.808	0.02	0.266	31.569	0.01	0.238	37.476	0.02	0.203	38.252	0.06	0.285	52.504	0.03	0.361	58.349	0.08
Immediate Recall (PRMPCI)	−0.176	2.984	0.03	−0.201	3.125	0.02	−0.271	3.840	0.01	−0.292	4.034	0.01	−0.047	4.760	0.71	−0.083	5.350	0.6
Delayed Recall (PRMPCD)	−0.213	3.187	0.01	−0.255	3.316	0.01	−0.247	4.352	0.02	−0.261	4.578	0.02	−0.176	4.685	0.16	−0.275	5.267	0.05
Planning Efficiency (OTSPSFC)	−0.416	0.553	0.01	−0.453	0.581	0.01	−0.389	0.751	0.01	−0.410	0.756	0.01	−0.460	0.826	0.01	−0.554	0.920	0.01
Planning Latency (OTSMDLFC)	−0.93	1194.021	0.26	−0.098	1231.321	0.25	−0.126	1616.025	0.25	−0.150	1699.866	0.20	−0.048	1799.830	0.70	−0.92	1907.740	0.49
Spatial Working Memory Errors (SWMBE468)	0.105	1.625	0.20	0.142	1.693	0.09	0.134	2.198	0.22	0.142	2.350	0.22	0.069	2.446	0.59	0.169	2.658	0.21
Strategy Use (SWMS)	0.085	0.371	0.30	0.085	0.389	0.32	0.030	0.503	0.78	0.026	0.528	0.82	0.160	0.557	0.20	0.179	0.629	0.20
Spatial Memory Errors (SWMBE4)	0.126	0.229	0.13	0.149	0.237	0.07	0.116	0.290	0.29	0.116	0.302	0.29	0.147	0.528	0.24	0.225	0.405	0.10
Spatial Memory Errors (SWMBE6)	0.109	0.603	0.18	0.123	0.635	0.15	0.138	0.779	0.21	0.083	0.810	0.41	0.075	0.075	0.55	0.159	1.060	0.24
Spatial Memory Errors (SWMBE8)	0.065	1.156	0.43	0.107	1.199	0.21	0.096	1.620	0.38	0.124	1.739	0.29	0.024	1.639	0.85	0.106	1.794	0.43

* Adjusted for gender, age, education, income, employment, governorate, and marital status.

Table 5 presents the standardized β coefficients from the adjusted regression model ranked by relative importance. The strongest associations with Hikikomori status were observed for visual processing accuracy (RVPA) and planning efficiency (OTSPSFC), which showed comparable standardized effect sizes (β ≈ −0.45, p = 0.001 for both). These were followed by response latency (RVPMDL) (β = 0.266, p = 0.001), delayed recall (PRMPCD) (β = −0.255, p = 0.003), and immediate recall (PRMPCI) (β = −0.201, p = 0.019). All remaining cognitive domains showed non-significant association.

**Table 5.** Ranking of CANTAB cognitive outcomes by relative importance (standardized β).

Rank	Cognitive Domain	CANTAB Measure	Standardized β	p-value	Effect Direction
1	Visual Processing Accuracy	RVPA	−0.453	0.001	↓ Hikikomori
2	Planning Efficiency	OTSPSFC	−0.453	0.001	↓ Hikikomori
3	Delayed Recall	PRMPCD	−0.255	0.003	↓ Hikikomori
4	Immediate Recall	PRMPCI	−0.201	0.019	↓ Hikikomori
5	Response Latency	RVPMDL	+0.266	0.001	↑ (Slower in Hikikomori)
6	Planning Latency	OTSMDLFC	−0.098	0.25	↔ Non-significant
7	Spatial Working Memory Errors	SWMBE468	+0.142	0.09	↔ Non-significant
8	Strategy Use	SWMS	+0.085	0.32	↔ Non-significant
9	Spatial Memory Errors (4 boxes)	SWMBE4	+0.149	0.07	↔ Non-significant
10	Spatial Memory Errors (6 boxes)	SWMBE6	+0.123	0.15	↔ Non-significant
11	Spatial Memory Errors (8 boxes)	SWMBE8	+0.107	0.21	↔ Non-significant

(Values are standardized β coefficients from the adjusted regression model, ranked by absolute magnitude. Negative β reflects poorer cognitive performance among Hikikomori cases).

## Discussion

The present study examined neurocognitive performance using cases identified through structured clinical assessment. Hikikomori has been extensively studied in East Asia, yet its cognitive aspects remain unexamined in the Middle East, leaving a significant literature gap. As cultural and societal factors influence its presentation, investigating Hikikomori in Oman is essential for developing region-specific diagnostic frameworks and interventions.

This study assesses cognitive performance in Hikikomori cases and healthy controls, with a focus on executive function, memory recall, and processing speed. Given the scarcity of research in the region, it also examines socio-demographic characteristics, offering insights into Hikikomori within an Omani context. As the first study to examine cognitive functioning in Hikikomori individuals using CANTAB, this research provides novel insights into neurocognitive risk factors while addressing a critical gap in Middle Eastern literature.

### Executive function and processing speed

Our results indicate that Hikikomori individuals experience executive function deficits, particularly in planning efficiency and response inhibition, which suggests difficulties in cognitive flexibility, decision-making, and problem-solving. These impairments are well-documented in psychiatric conditions such as schizophrenia, bipolar disorder, and anxiety disorders ( Bourke et al., 2012; Gau & Shang, 2010), which research has found are comorbid in individuals with Hikikomori ( Cai et al., 2023; Chauliac et al., 2017). CANTAB has been established as a reliable tool for assessing executive function ( Luciana, 2003). Prior research indicates that deficits in response inhibition and attentional shifting, commonly seen in social anxiety disorder, may contribute to social withdrawal ( Pupi et al., 2025; Rhodes et al., 2005). Given the high prevalence of social anxiety in Hikikomori ( Kato et al., 2024), these cognitive difficulties may reinforce avoidance behaviors ( Cai et al., 2023; Lo et al., 2023). In addition to deficits in planning efficiency, Hikikomori cases also exhibited slower response times, suggesting broader impairments in processing speed and cognitive efficiency that may further exacerbate withdrawal tendencies.

In this study, planning efficiency and visual processing accuracy showed the strongest associations with Hikikomori, reflecting substantial impairments in goal-directed behavior and in the interpretation of environmental cues necessary for social interaction. These findings are consistent with evidence showing that executive dysfunction and attentional processing difficulties are linked to reduced social functioning and withdrawal in clinical populations ( Bourke et al., 2012; Kim et al., 2014; Luciana & Nelson, 2002). The minimal associations observed for spatial working memory and strategy use suggest that Hikikomori is characterized by selective rather than global cognitive impairment, a pattern also reported in recent neuropsychological research on Hikikomori ( Santona et al., 2023) and in studies demonstrating that only specific executive domains may be affected rather than overall cognitive capacity ( Rhodes et al., 2005). This selective vulnerability highlights the importance of interventions that target planning, cognitive flexibility, and visual–attentional processing, rather than assuming a broad cognitive decline among Hikikomori individuals.

### Memory recall deficits

Our study showed that Hikikomori individuals exhibited impairments in immediate and delayed memory recall, suggesting difficulties in processing and retrieving information, potentially affecting academic performance, work responsibilities, and social interactions. Memory deficits have been observed in individuals with social anxiety disorder ( Airaksinen et al., 2005), and dysfunction in the medial temporal lobe and prefrontal cortex has been linked to social cognition impairments ( Luciana & Nelson, 2002). Given that Hikikomori individuals often display heightened sensitivity to social judgment ( Sakamoto et al., 2005), difficulties in retaining and recalling social information may reinforce withdrawal tendencies ( Kubo et al., 2022).

### Visual processing and attention

We found that Hikikomori individuals demonstrated significant deficits in visual processing accuracy, after adjusting for socio-demographic variables, suggesting a specific vulnerability in interpreting environmental cues critical for social engagement. In contrast, no significant differences were observed in spatial working memory errors, planning latency, or strategy use, indicating that Hikikomori does not reflect global cognitive dysfunction. These findings align with research showing that psychiatric conditions often affect distinct cognitive domains ( Bourke et al., 2012; Kim et al., 2014; Luciana & Nelson, 2002).

Given that social anxiety disorder (SAD) is one of the most common comorbidities in Hikikomori ( Lin et al., 2022; Pupi et al., 2025; Tateno et al., 2012), it is plausible that attentional and cognitive flexibility impairments—key features of SAD—may contribute to persistent withdrawal behaviors. SAD has been linked to difficulties in processing social cues and regulating attention and emotion ( Bourke et al., 2012), which may explain the heightened social disengagement observed in Hikikomori cases ( Bowker et al., 2019; Chauliac et al., 2017).

Furthermore, neuroimaging studies have shown that chronic social withdrawal is associated with changes in brain regions responsible for visual processing and attention, including the hippocampus, ventral striatum, and prefrontal cortex ( Zovetti et al., 2021). These findings align with recent work on COVID-19-related isolation, which underscores the broader neurological and psychological consequences of prolonged social disengagement ( Roza et al., 2020).

### Spatial working memory and strategy use

This study demonstrated that Hikikomori individuals showed deficits in executive function, memory recall, and attention, while their spatial working memory remained intact. This suggests that not all cognitive domains are equally affected ( Luciana & Nelson, 2002). While spatial memory was preserved, impairments in attention shifting and planning may still interfere with social and occupational functioning ( Rhodes et al., 2005). These findings highlight the importance of targeted interventions addressing executive function deficits, rather than assuming global cognitive impairments in Hikikomori individuals ( Santona et al., 2023). Furthermore, subgroup analyses among patients and attendees revealed consistent impairments in visual processing accuracy and planning efficiency, highlighting the robustness of these findings across different clinical and non-clinical populations ( Bowker et al., 2019; Siedlecki et al., 2019) .

### Gender-based differences

While previous research has established gender-based cognitive differences in the general population ( Camarata & Woodcock, 2006; Herlitz & Rehnman, 2008), our study did not find significant gender differences in memory recall or executive function among Hikikomori individuals. This suggests that social withdrawal may affect both genders similarly. Previous neuropsychological research also indicates that cognitive impairments in psychiatric conditions are not necessarily gender-dependent ( Grissom & Reyes, 2019). Although our study did not observe normative gender trends, previous research in the general population has shown that females often perform better in tasks involving attentional control and inhibition, while males tend to show advantages in spatial processing speed ( Giofre et al., 2024; Siedlecki et al., 2019). These normative trends may serve as a reference point for future studies exploring whether prolonged social withdrawal disrupts or attenuates gender-based cognitive patterns.

### Socioeconomic factors and cognitive performance

Our analysis revealed that Hikikomori individuals were more likely to be unemployed, consistent with research linking long-term social withdrawal to reduced workforce participation ( Nonaka & Sakai, 2021). While marital status and income level were not statistically different, Hikikomori cases tended to report lower income, suggesting that economic stressors may contribute to withdrawal behaviors. The role of socioeconomic barriers in Hikikomori highlights the need for interventions that address both cognitive and environmental factors. Cognitive disparities may also intersect with broader sociocultural influences, such as educational and occupational pathways shaped by gender-role identification and self-efficacy beliefs ( Nakajima et al., 2019; Riley et al., 2016). These factors could influence the cognitive reserves of individuals at risk for social withdrawal, compounding the effects of isolation and further limiting re-engagement with work or education.

### Implications for intervention

Given the overlap between Hikikomori and social anxiety disorder, interventions focusing on cognitive remediation may also help reduce avoidance behaviors and improve social engagement ( Bourke et al., 2012; Kato et al., 2024). Given known cognitive gender differences in verbal memory, inhibition, and spatial ability in the general population ( Goel & Tripathi, 2024; Riley et al., 2016), future research should examine whether targeted interventions can be tailored to individual cognitive strengths and weaknesses across genders within Hikikomori populations.

### Limitations

Several limitations should be considered. First, the case–control design precludes causal inference; cognitive differences may precede social withdrawal or emerge because of prolonged disengagement. Longitudinal studies are needed to clarify directionality. Second, multiple cognitive domains were examined, raising the possibility of type I error due to multiple comparisons. Although results showed a coherent pattern across key domains, findings should be interpreted cautiously. Third, participants were recruited from a hospital-based behavioral medicine clinic, introducing potential selection bias. While inclusion of non-patient attendees broadened representation, results may not fully generalize to community samples. Fourth, although assessors administering CANTAB were blinded to case status, psychiatric comorbidities—particularly depression and anxiety—were not statistically controlled in the cognitive analyses. As such, observed differences may reflect comorbidity-related cognitive effects rather than Hikikomori-specific mechanisms. Finally, medication status was not systematically assessed. Psychotropic medications may influence attention, processing speed, and memory, representing a potential confound.

Future research should integrate neuroimaging techniques to identify neural correlates of Hikikomori-related cognitive dysfunction, examine cultural variations across populations ( Kubo et al., 2022), and distinguish between primary Hikikomori and cases with psychiatric comorbidities. Given the high rates of co-occurring conditions such as social anxiety disorder, depression, and personality disorders among Hikikomori cases ( Teo & Gaw, 2010), it remains unclear whether the observed cognitive deficits are specific to Hikikomori itself.

## Conclusion

This study demonstrates that Hikikomori in Oman is associated with selective neurocognitive differences, particularly in planning efficiency, sustained attentional accuracy, processing speed, and visual memory recall. These findings extend existing Hikikomori research into a Middle Eastern context and suggest that cognitive inefficiencies may accompany prolonged social withdrawal.

Rather than indicating global cognitive decline, the results point to specific domains that may contribute to functional disengagement. Future longitudinal and multimodal studies are needed to clarify causality, disentangle comorbidity effects, and determine whether these cognitive patterns change with recovery or intervention.

## Ethics approval and consent to participate

Ethical approval was obtained from the Sultan Qaboos University Hospital Ethics Committee (MREC #2260). Written informed consent was obtained from all participants.

## Availability of data and materials

The datasets generated and/or analyzed during the current study are not publicly available due to ethical and confidentiality restrictions imposed by the Sultan Qaboos University Hospital Ethics Committee (MREC #2260), as the data contain sensitive clinical and potentially identifiable participant information.

The Ethics Committee does not permit open public sharing of individual-level clinical data; however, data may be made available upon reasonable request for academic and research purposes.

Researchers who wish to access the data must submit a formal request outlining the purpose of use and intended analyses. Requests are subject to approval by the corresponding author and, where applicable, the Sultan Qaboos University Hospital Ethics Committee.

Requests for data access can be directed to:

Professor Yahya M. Al-Farsi (Corresponding Author).

Email: ymfarsi@squ.edu.om

Access will be granted under conditions that ensure compliance with ethical guidelines, data protection regulations, and participant confidentiality.

## Authors’ information

NAK is a PhD candidate in Public Health and Epidemiology in Sultan Qaboos University, Oman.
