# Executive Dysfunction and Memory Impairments in Hikikomori: A Neuropsychological Case-Control Study from Oman

**DOI:** 10.12688/f1000research.181828.4

**Published:** 2026-09-22

**Authors:** Nutaila Al Kharusi, Yahya M. Al-Farsi, Moon Fai Chan, Samir Al-Adawi, Mallak Al-Mandhari, Nasser Al-Sibani

**Affiliations:** 1 Department of Family Medicine & Public Health, Sultan Qaboos University College of Medicine and Health Science, Muscat, Muscat Governorate, 123, Oman; 2 Behavioral Medicine, Sultan Qaboos University College of Medicine and Health Science, Muscat, Muscat Governorate, 123, Oman

**Keywords:** Hikikomori, Social Withdrawal, Case-Control Study, Neurocognition, CANTAB, Executive Function, Memory Recall, Oman.

## Abstract

**Background:**

Hikikomori, a form of prolonged social withdrawal, has been widely studied in East Asia but remains underexplored in the Middle East. Cultural and social factors may influence its cognitive profile, necessitating localized research.

**Aim:**

To compare cognitive performance between Hikikomori cases and non-cases in Oman using the Cambridge Neuropsychological Test Automated Battery (CANTAB), focusing on executive function, memory recall, and processing speed.

**Methods:**

A case-control study was conducted in 2024 with 150 participants (54 Hikikomori cases, 96 controls) recruited from a tertiary hospital in Oman. Hikikomori cases were classified using a broad HiDE-I definition, which included pathological, at-risk, and resemblance Hikikomori categories. CANTAB was used to assess key cognitive domains. Independent t-tests and linear regression analyses were performed to compare outcomes.

**Results:**

Hikikomori cases showed significant impairments in visual processing accuracy (p < 0.001), response latency (p = 0.004), immediate recall (p = 0.031), delayed recall (p = 0.009), and planning efficiency (p < 0.001) compared to non-cases. Adjusted regression analyses—controlling for age, gender, education, income, employment, marital status, and governorate—confirmed that Hikikomori status was significantly associated with lower visual processing accuracy (β = −0.453, p = 0.001), slower response latency (β = 0.266, p = 0.001), poorer immediate (β = −0.201, p = 0.019) and delayed recall (β = −0.255, p = 0.003), and reduced planning efficiency (β = −0.453, p = 0.001). No significant differences were found for spatial working memory tasks.

**Conclusion:**

Hikikomori individuals in Oman exhibit significant deficits in executive function, memory recall, processing speed, and visual attention, even after adjusting for socio-demographic factors. These findings underscore the importance of early neurocognitive screening and tailored interventions. This is one of the first studies to explore Hikikomori’s cognitive profile in the Middle East.

**List of abbreviations**
B/β: Beta Coefficient
CANTAB: Cambridge Neuropsychological Test Automated Battery
CI: Confidence Interval
HiDE-I : Hikikomori Diagnostic Evaluation Interview
HLID: Hikikomori-like Idiom of Distress
IED: Intra/Extra Dimensional Set Shift
OTSMDLFC: One Touch Stockings of Cambridge – Mean Latency to First Choice
OTSPSFC: One Touch Stockings of Cambridge – Problems Solved on First Choice
PRMPCI: Pattern Recognition Memory – Percent Correct Immediate
PRMPCD: Pattern Recognition Memory – Percent Correct Delayed
RVPA: Rapid Visual Processing Accuracy
RVPMDL: Rapid Visual Processing Mean Response Latency
SAD: Social Anxiety Disorder
SQUH: Sultan Qaboos University Hospital
SSP: Spatial Span
SWMBE4: Spatial Working Memory – Between Errors (4 Boxes)
SWMBE6: Spatial Working Memory – Between Errors (6 Boxes)
SWMBE8: Spatial Working Memory – Between Errors (8 Boxes)
SWMBE468: Spatial Working Memory – Between Errors (4, 6, 8 Boxes Combined)
SWMS: Spatial Working Memory – Strategy Score
SD: Standard Deviation
SE: Standard Error
SPSS: Statistical Package for the Social Sciences

## Introduction

Hikikomori, a severe form of social withdrawal where individuals isolate themselves for extended periods avoiding school, work, and social interactions, was initially documented in Japan and has since been recognized globally, including in the Middle East ( Kato et al., 2019; Kato et al., 2020, 2024; Saito, 1998; Tan et al., 2021; Teo et al., 2023). Hikikomori has been widely documented as comorbid with psychiatric disorders such as social anxiety disorder (SAD) and depression ( Kato et al., 2019; Koyama et al., 2010; Teo et al., 2020). These disorders are themselves characterized by cognitive impairments — including deficits in executive function, attentional control, and working memory — that may further reinforce social withdrawal ( Airaksinen et al., 2005; Bourke et al., 2012; Kim et al., 2014; Luciana, 2003; Rhodes et al., 2005).

In Oman, a high-income Arab country with rapid social changes and evolving family structures, Hikikomori-like behaviors are emerging amid increasing academic and career pressures ( Al-Sibani et al., 2023; Sakamoto et al., 2005). Despite a population of approximately 5.1 million as of 2023 ( World Bank, 2023), mental health services are underutilized due to stigma and limited awareness, potentially obscuring the true prevalence of Hikikomori in the region ( Al Alawi et al., 2017; Albadi et al., 2025). Recent findings in Oman indicate significant subgroups exhibiting Hikikomori-like social withdrawal ( Al-Sibani et al., 2023; Chan et al., 2024). While earlier work operationalized this presentation as Hikikomori-like idiom of distress (HLID), the present study focuses specifically on clinically classified Hikikomori cases defined using the HiDE-I. Because self-report screening measures such as the HQ-25 can misclassify cases at the margins of any cut-off, this study used the Hikikomori Diagnostic Evaluation Interview (HiDE-I), a structured clinician-administered interview, to confirm case status. In a companion diagnostic-performance analysis on this cohort, no single HQ-25 threshold achieved simultaneously high sensitivity and specificity against HiDE-I classification, supporting the use of a clinician-administered interview as the reference standard for case ascertainment in the present cognitive comparison.

Research shows that social withdrawal in Hikikomori is associated with psychiatric comorbidities such as depression, anxiety, and avoidant personality disorder ( Bowker et al., 2019; Chauliac et al., 2017; Koyama et al., 2010; Teo et al., 2020). A survey found that 54.5% of those meeting Hikikomori criteria had a lifetime psychiatric disorder, with anxiety and depressive disorders being the most common ( Koyama et al., 2010).

From a neurocognitive perspective, we hypothesized that individuals with Hikikomori would demonstrate impairments primarily in executive and attentional domains—particularly planning efficiency, processing speed, and memory recall—rather than in basic spatial working memory. These domains are consistently implicated in depression, anxiety, and avoidant psychopathology, which frequently co-occur with Hikikomori. In depression, a meta-analysis using the same CANTAB battery employed here found moderate deficits in executive function, memory, and attention relative to non-cases (Cohen’s d approximately −0.34 to −0.65), with comparable deficits persisting even after symptom remission (Rock et al., 2014). In contrast, spatial working memory deficits were not expected to be a core feature of Hikikomori itself but may emerge secondarily in more severe or clinically complex presentations.

Given Oman’s collectivist culture and rapid modernization, Hikikomori-like social withdrawal is emerging as a culture-reactive phenomenon rather than one unique to Japan ( Al-Sibani et al., 2023). Al-Sibani et al. (2023) reported that 44% of participants exhibited HLID following the COVID-19 pandemic, with risk factors such as screen overuse, unemployment, mental illness, and adverse childhood experiences, echoing global Hikikomori patterns. These findings align with regional data from the United Arab Emirates, where problematic social media use, gaming behavior, and loneliness were significantly associated with Hikikomori-like traits, suggesting digital over-engagement as a potential pathway into social withdrawal ( Shah et al., 2024).

To explore cognitive impairments associated with this condition, this study employed the Cambridge Neuropsychological Test Automated Battery (CANTAB)—a non-verbal, examiner-independent tool that minimizes cultural and language biases ( Chamberlain et al., 2011; Fray et al., 1996; Luciana, 2003; Mehta et al., 2004; Rhodes et al., 2005). Understanding whether Hikikomori carries a specific cognitive signature — beyond the emotional distress that is already well documented — matters clinically: if cognitive impairment is present and is not simply a downstream consequence of comorbid depression or anxiety, standard psychiatric treatment targeting mood and anxiety symptoms alone may be insufficient, and interventions may need to separately address planning, attention, and memory difficulties that interfere with re-engagement in work, education, and social life.

This study aims to compare the cognitive performance of Hikikomori cases and healthy non-cases in Oman, using neuropsychological assessments to identify deficits in executive function, memory recall, and processing speed. Given the lack of research on Hikikomori in the Middle East, this study also seeks to examine socio-demographic characteristics of Hikikomori cases versus non-cases among attendees of the Behavioral Medicine Clinic in Oman. By comparing cognitive performance using CANTAB, this research will provide insights into potential neurocognitive risk factors associated with Hikikomori and contribute to the understanding of social withdrawal syndromes within the Omani context.

To our knowledge, this is the first study to examine Hikikomori individuals using CANTAB in the Middle East. This study builds on prior work evaluating the diagnostic performance of the HQ-25 against the clinician-administered HiDE-I in Oman and uses HiDE-I–based case classification to examine whether individuals with Hikikomori also show measurable neurocognitive differences. This allows the analysis to move beyond screening-level identification and focus on cognitively relevant differences among clinically classified cases.

## Methods

### Study design and setting

This case–control study was conducted at the Behavioral Medicine Department of Sultan Qaboos University Hospital (SQUH), a tertiary care teaching hospital in Muscat, Oman. The study was nested within a larger cross-sectional investigation that aimed to identify Hikikomori-like idiom of distress (HLID) among adults attending the department. Initial screening was carried out using the Hikikomori Questionnaire-25 (HQ-25), alongside the collection of socio-demographic information. Participants were subsequently invited to complete a structured diagnostic interview using the HiDE-I to allow for accurate case classification. Participants who completed the HiDE-I and met eligibility criteria for the CANTAB sub-study were invited to complete neuropsychological testing. Selection was non-random and depended on participant consent and logistical feasibility within the clinical setting. The resulting CANTAB group should therefore be regarded as a convenience subsample rather than a representative random sample of all 454 interviewed participants. Hikikomori case status in the present study was determined using the clinician-administered HiDE-I rather than HQ-25 screening scores alone.

### Participants

The initial cross-sectional sample consisted of 1,200 Omani nationals aged between 18 and 59 years, recruited from outpatients attending the Behavioral Medicine Department at SQUH and their accompanying attendees. Of these, 454 participants completed the HiDE-I structured diagnostic interview. The HiDE-I was incorporated into the study protocol from 1 January 2024 onward, following its publication ( Teo et al., 2023); participants recruited before this date completed HQ-25 screening only, and from 1 January 2024 onward all eligible attendees were invited to complete the HiDE-I irrespective of HQ-25 score. From this group, 150 participants were selected to complete CANTAB testing, including 85 patients and 65 attendees. The 150 participants who completed CANTAB were selected from the 454 HiDE-I-interviewed participants by convenience sampling, prioritizing available HiDE-I-positive cases to maximize case numbers within the resource and scheduling constraints of CANTAB administration (equipment availability and staff time), with non-cases recruited concurrently from the same clinic population. Participants were not randomly or systematically sampled from the full interviewed cohort, which may limit representativeness of the CANTAB sub-sample relative to the broader 454-participant group; sociodemographic comparability between the two is presented in  Table 2. Cognitive performance outcomes were examined across multiple domains. The importance of each cognitive domain was evaluated using standardized β coefficients derived from adjusted regression models (  Figure 1).

**Figure 1.**
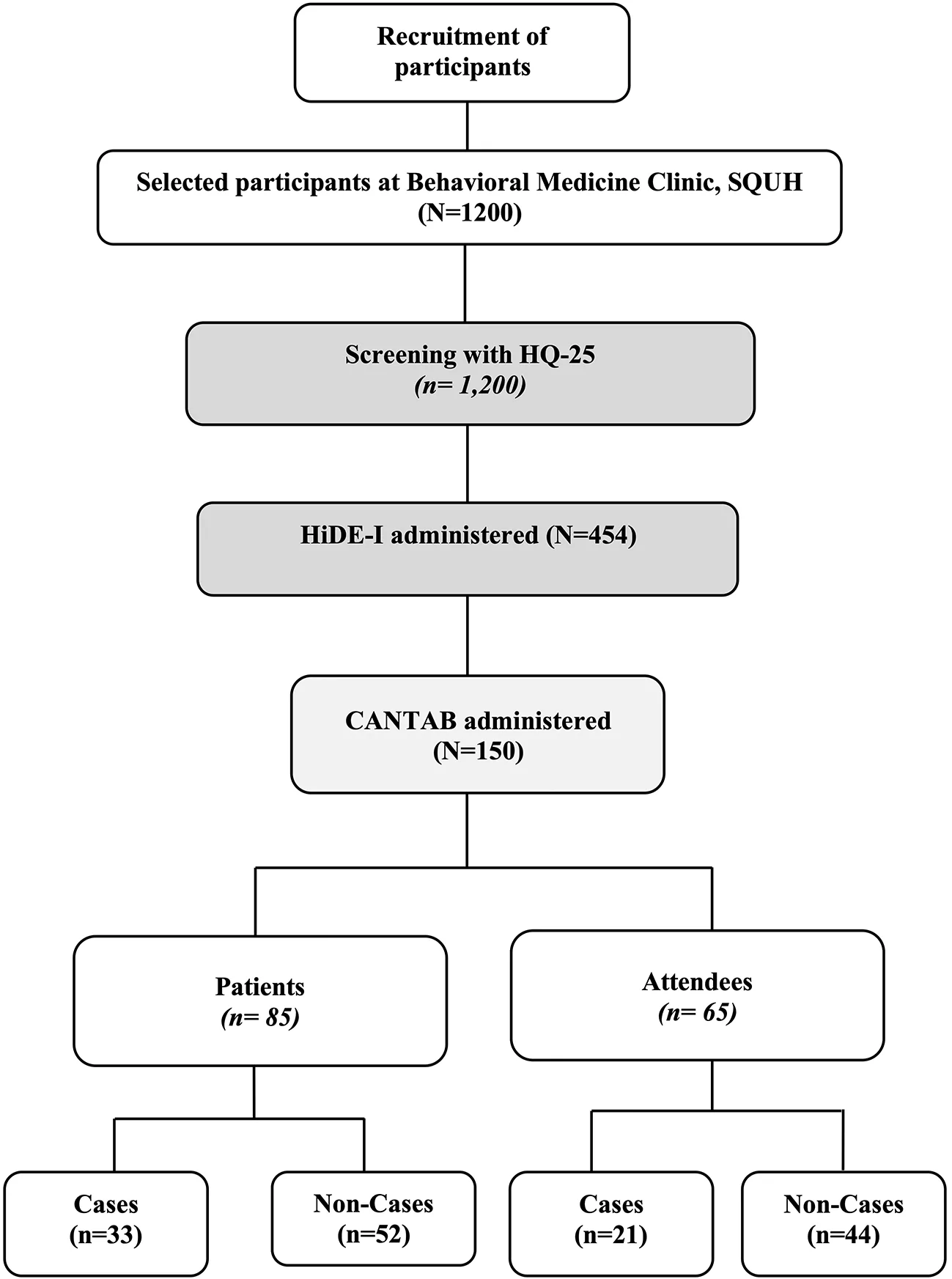
Recruitment and classification of participants at the Behavioral Medicine Clinic, SQUH, Oman, 2024.

### Case ascertainment & selection of cases

Hikikomori cases were identified using the Hikikomori Diagnostic Evaluation Interview (HiDE-I), a structured clinical assessment tool that aligns with international diagnostic criteria for prolonged social withdrawal ( Teo et al., 2023).

The HiDE-I is a structured clinician-administered interview assessing the frequency and purpose of outings, duration of withdrawal, social participation and interaction, and distress, concern by others, or functional impairment associated with withdrawal. For this neurocognitive study, participants classified as pathological, at-risk, or resembling Hikikomori were included as HiDE-I-positive cases, irrespective of HQ-25 score; participants meeting none of these categories were classified as non-cases. The HiDE-I classifies respondents through three sequential criteria ( Teo et al., 2023). Criterion I (physical withdrawal): during the preceding month, the respondent spent three days or fewer per week engaged in an activity outside the home lasting at least one hour. Criterion II (distress and impairment): the withdrawal is associated with distress or loneliness in the respondent, concern or help-seeking by family members, or impairment in occupational or academic functioning, home life, or friendships, assessed through the corresponding HiDE-I item sets. Criterion III (duration): the length of time the respondent has been physically withdrawn. Respondents not meeting Criterion I were classified as non-cases. Respondents meeting Criterion I but not Criterion II were classified as Hikikomori resemblance. Respondents meeting Criteria I and II were classified by duration of withdrawal as pathological Hikikomori (six months or more) or at-risk Hikikomori (less than six months). The HiDE algorithm further subdivides the under-six-month group into at-risk (less than three months) and pre-pathological (three to less than six months); for this study these two bands were combined into a single at-risk category, as neither meets the six-month duration threshold for pathological Hikikomori. Participants were therefore classified into four groups: non-case, Hikikomori resemblance, at-risk Hikikomori, and pathological Hikikomori.

For the purposes of this neurocognitive case-control study, a broad HiDE-I case definition was used. This definition classified participants as Hikikomori cases if they met criteria for any HiDE-I category: pathological Hikikomori, at-risk Hikikomori, or Hikikomori resemblance. Participants who did not meet criteria for any of these three categories were classified as non-cases. This broader definition was deliberately adopted because cognitive differences associated with social withdrawal may not be limited to fully pathological Hikikomori but may also be present in earlier or subthreshold stages of withdrawal.

A total of 54 participants were identified as Hikikomori cases and agreed to participate in the study, yielding a response rate of 93%. All cases were evaluated by two trained mental health professionals, each experienced in assessing social withdrawal disorders. Inter-rater reliability was assessed using percent agreement between two independent clinicians who conducted the HiDE-I interviews. Initial agreement on Hikikomori classification across the three subtypes was 96%. Discrepant cases were subsequently reviewed through a structured consensus adjudication process involving joint discussion of interview data and clinical criteria, after which full agreement (100%) was reached. This consensus-based approach was used to ensure diagnostic accuracy rather than to inflate reliability estimates.

### Selection of Non-cases

Non-case participants were recruited from the Behavioral Medicine Department at SQUH and confirmed to be Hikikomori-free through the Hikikomori Diagnostic Evaluation Interview (HiDE-I). To be included, participants were required to have no history of prolonged social withdrawal and not meet criteria for any HiDE-I subcategory, including pathological, at-risk, or resemblance Hikikomori. A total of 96 non- cases consented to participate, yielding a response rate of 87%.

Inter-rater reliability for non-case classification was assessed using percent agreement between two independent clinicians who conducted the HiDE-I interviews. Initial agreement on control status was 95%. Discrepant cases were subsequently reviewed through a structured consensus adjudication process involving joint discussion of interview data and clinical criteria, after which full agreement (100%) was reached. This consensus approach was applied to ensure accurate exclusion of Hikikomori rather than to inflate reliability estimates.

### Study tools

Hikikomori cases and non-cases were assessed using standardized screening and diagnostic tools. The Arabic validated version of the Hikikomori Questionnaire (HQ-25), which has demonstrated excellent internal consistency (Cronbach’s alpha = 0.91), was used to assess social withdrawal ( Al-Sibani et al., 2023). Sociodemographic data were also collected using a structured questionnaire administered in the previous cross-sectional study, providing initial data on social withdrawal patterns and participant characteristics. Participants completed a structured self-report sociodemographic questionnaire covering age, gender, marital status, education, monthly household income, employment status, governorate of residence, and whether they had children. For the present analysis, age was grouped as 18–29, 30–39, and ≥40 years; income as <1,000, 1,000–1,999, 2,000–2,999, and ≥3,000 OMR; employment as employed or unemployed; marital status as never married or ever married; and education as <12, 12, or >12 years. Governorates represented in the sample are listed in  Table 2. In addition to Hikikomori-specific assessments, participants underwent cognitive testing using the Cambridge Neuropsychological Test Automated Battery (CANTAB) ( Fray et al., 1996). CANTAB assesses core domains such as executive function, memory, attention, and response inhibition, and has been validated in both clinical and non-clinical populations across diverse cultural contexts ( Airaksinen et al., 2005; Bourke et al., 2012; Green et al., 2019; Kim et al., 2014).

CANTAB is a computerized neuropsychological assessment tool widely used in psychiatric research to measure executive function, attention, working memory, and processing speed. Testing was conducted via touchscreen devices, with responses recorded automatically. A trained examiner provided standardized instructions to ensure comprehension. The testing was conducted in a quiet environment, and headphones were provided when necessary to minimize auditory distractions.

CANTAB assessed multiple cognitive domains relevant to this study. Executive function was measured using the Intra/Extra Dimensional Set Shift (IED), which evaluates cognitive flexibility, and the One Touch Stockings of Cambridge (OTS), which assesses planning and problem-solving skills. Working memory was tested using the Spatial Working Memory (SWM) and Spatial Span (SSP) tasks. Attention and processing speed were assessed through the Rapid Visual Information Processing (RVP) task, while Pattern Recognition Memory (PRM) was used to evaluate visual memory recall. The CANTAB tasks and corresponding outcome measures used in this study are summarized in Table 1.

**Table 1.** CANTAB Tasks and outcome measures used in the cognitive assessment.

Cognitive domain	CANTAB task	Outcome variable (used in analyses)	Description
Visual attention & processing speed	Rapid Visual Information Processing (RVP)	RVPA RVPMDL	Proportion of correct detections (visual processing accuracy) Mean response latency (processing speed)
Visual memory	Pattern Recognition Memory (PRM)	PRMPCI PRMPCD	Percentage correct on immediate recall Percentage correct on delayed recall
Executive function (planning)	One Touch Stockings of Cambridge (OTS)	OTSPSFC OTSMDLFC	Number of problems solved on first choice (planning efficiency) Mean latency to first choice (planning latency)
Working memory	Spatial Working Memory (SWM)	SWMBE468 SWMBE4 SWMBE6 SWMBE8	Total between-search errors (all box sizes) Errors at 4-box stage Errors at 6-box stage Errors at 8-box stage
Strategy use	Spatial Working Memory (SWM)	SWMS	Strategy score (lower scores indicate more efficient strategy use)

### Data collection

Data was collected through standardized interviews and computerized cognitive assessments. Trained mental health professionals administered all assessments following standardized protocols. HiDE-I interviews took place in private clinical settings, and CANTAB assessments were conducted in a controlled environment with sufficient time for task completion. Assessors administering the CANTAB were blinded to participants’ Hikikomori case or control status to reduce the risk of performance and observer bias.

### Statistical analysis

Data were analyzed using SPSS version 27.0 (IBM Corp., Armonk, NY, USA). Descriptive statistics were used to summarize participant characteristics, with means and standard deviations reported for continuous variables and frequencies with percentages for categorical variables. Independent samples t-tests were conducted to compare cognitive performance between Hikikomori cases and non-cases. Associations between Hikikomori status and cognitive performance were examined using linear regression models, including both crude models and adjusted models controlling for age, gender, education level, income, employment status, governorate, and marital status. Regression assumptions were assessed prior to analysis. Normality of residuals was evaluated using visual inspection of histograms and Q-Q plots, while homoscedasticity and linearity were assessed through residual scatterplots. Reaction time and latency measures, which showed mild positive skew, were retained in their original scale, as residual diagnostics indicated acceptable approximation to normality and model fit. No variable transformations were required. To ensure robustness of findings, non-parametric sensitivity analyses were also performed, including Mann–Whitney U tests for group comparisons and Spearman’s rank correlation coefficients for associations. Results from these supplementary analyses were consistent with the parametric findings.

Sample size for the broader parent case–control study was calculated a priori using Rothman’s Episheet and was based on odds-ratio detection, assuming a control-to-case ratio of 1:1, a Zα value of 1.96, an exposure prevalence of 0.15, and 80% power to detect an odds ratio of 2.0. This calculation was not specifically tailored to the continuous CANTAB outcomes examined in the present neurocognitive sub-study. A two-group calculation based on an anticipated mean difference and variance would have been more directly appropriate.

The achieved case-to-non-case ratio was 54:96 (approximately 1:1.8), rather than the 1:1 ratio assumed in the parent-study calculation. With a fixed total sample size and similar group variances, unequal allocation generally provides less precision than equal allocation; however, recruiting additional non-cases can improve precision when the number of available cases is constrained. Because the original calculation was not based on the continuous CANTAB outcomes, the power implications of the achieved ratio were not quantified. The study may therefore have had limited precision for detecting small effects, and non-significant findings should not be interpreted as evidence of equivalence.

Given that 11 cognitive outcomes were examined, we additionally applied Benjamini–Hochberg false-discovery-rate correction (q = 0.05) to the adjusted-model p-values in  Table 5. All five domains reported as significant in the primary analysis remained significant after correction. Under the more conservative Bonferroni correction (α = 0.0045), four of the five domains remained significant; immediate recall (p = 0.019) did not survive this stricter threshold and should be interpreted with additional caution. These results indicate that the pattern of findings is unlikely to be attributable to multiple testing alone, though replication in an independent, adequately powered sample remains warranted.

## Results

### Socio-demographic characteristics Hikikomori cases and non-cases 

Table 2 presents the socio-demographic characteristics of participants who underwent CANTAB testing. Of the 150 participants included, 54 were classified as Hikikomori cases and 96 as non-cases based on the study’s HiDE-I-positive case definition, which included pathological, at-risk, and resembling presentations irrespective of HQ-25 score. Patients constituted 56.7% of the sample (n = 85), while attendees accounted for 43.3% (n = 65).

**Table 2.** Socio-demographic characteristics of Hikikomori cases and non-cases undergoing CANTAB, Oman, 2024.

Characteristic	All participants (N = 150)		Patients (N = 85)		Attendees (N = 65)	
	Cases (N/%) 54	Non-cases (N/%) 96	Cases (N/%) 33	Non-cases (N/%) 52	Cases (N/%) 21	Non-cases (N/%) 44
**Gender**						
**Female**	38 (70.4)	60 (62.5)	25 (75.8)	37 (71.2)	13 (61.9)	23 (52.3)
Male	16 (29.6)	36 (37.5)	8 (9.4)	15 (17.6)	8 (12.3)	21 (32.3)
**Age categories**						
**18–29**	42 (77.8)	65 (67.7)	26 (30.6)	37 (43.5)	16 (24.6)	28 (43.1)
30–39	8 (14.8)	17 (17.7)	4 (4.7)	9 (10.6)	4 (6.2)	8 (12.3)
40 or more	4 (7.4)	14 (14.6)	3 (3.5)	6 (7.1)	1 (1.5)	8 (12.3)
**Family income**						
**<1000**	25 (46.3)	43 (44.8)	13 (15.3)	23 (27.1)	12 (18.5)	20 (30.8)
**1000–1999**	20 (37.0)	30 (31.2)	12 (14.1)	14 (16.5)	8 (12.3)	16 (24.6)
2000–2999	6 (11.1)	14 (14.6)	6 (7.1)	9 (10.6)	0 (0.0)	5 (7.7)
3000 or more	3 (5.6)	9 (9.4)	2 (2.4)	6 (7.1)	1 (1.5)	3 (4.6)
**Employment**						
Employed	8 (14.8)	31 (32.3)	6 (7.1)	14 (16.5)	2 (3.1)	17 (26.2)
**Unemployed**	46 (85.2)	65 (67.7)	27 (31.8)	38 (44.7)	19 (29.2)	27 (41.5)
**Marital Status**						
Never Married	39 (72.2)	70 (72.9)	23 (27.1)	42 (49.4)	16 (24.6)	28 (43.1)
Ever Married	15 (27.8)	26 (27.1)	10 (11.8)	10 (11.8)	5 (7.7)	16 (24.6)
**Have children**						
Yes	12 (22.2)	21 (21.9)	7 (21.2)	6 (11.5)	5 (23.8)	15 (34.1)
No	42 (77.8)	75 (78.1)	26 (78.8)	46 (88.5)	16 (76.2)	29 (65.9)
**Education years**						
Less than 12 years	0 (0.0)	4 (4.2)	0 (0.0)	0 (0.0)	0 (0.0)	4 (6.2)
12 years	16 (29.6)	25 (26.0)	9 (10.6)	12 (14.1)	7 (10.8)	13 (20.0)
Above 12 years	38 (70.4)	67 (69.8)	24 (28.2)	40 (47.1)	14 (21.5)	27 (41.5)
**Governorate**						
Buraimi	0 (0.0)	1 (1.0)	0 (0.0)	0 (0.0)	0 (0.0)	1 (1.5)
Dakhliya	10 (18.5)	10 (10.4)	4 (4.7)	3 (3.5)	6 (9.2)	7 (10.8)
Dhahirah	1 (1.9)	5 (5.2)	1 (1.2)	4 (4.7)	0 (0.0)	1 (1.5)
Sharqiyah	5 (9.3)	6 (6.2)	2 (2.4)	3 (3.5)	3 (4.6)	3 (4.6)
Batinah	17 (31.5)	21 (21.9)	11 (12.9)	9 (10.6)	6 (9.2)	12 (18.5)
Dhofar	1 (1.9)	0 (0.0)	1 (1.2)	0 (0.0)	0 (0.0)	0 (0.0)
Muscat	20 (37.0)	53 (55.2)	14(16.5)	33 (38.8)	6(9.2)	20 (30.8)

Values are presented as n (%). Percentages are calculated within column denominators (i.e., within cases and non-cases for all participants, patients, and attendees, respectively). Hikikomori case status was determined using the HiDE-I broad definition, including pathological, at-risk, and resemblance categories.

Among Hikikomori cases, the majority were female (70.4%) and aged 18–29 years (77.8%), compared with 62.5% and 67.7%, respectively, among non-cases. A higher proportion of cases were unemployed (85.2%) compared to non-cases (67.7%). Differences were also observed across family income categories, with cases more frequently represented in lower income brackets.

No substantial differences were observed between cases and non-cases with respect to marital status, parental status, educational attainment, or governorate of residence. Distributions across governorates reflected the clinic-based sample, with Muscat and Al Batinah accounting for the largest proportions in both groups.

### Cognitive performance comparison between Hikikomori cases and non-cases 

Table 3 presents comparisons of cognitive performance between Hikikomori cases and non-cases. Across the full sample, Hikikomori cases demonstrated significantly poorer performance in visual processing accuracy, response latency, immediate recall, delayed recall, and planning efficiency (p < 0.05). No significant group differences were observed for planning latency, spatial working memory errors, or strategy use.

**Table 3.** Comparison of cognitive performance metrics between Hikikomori cases and non-cases, Oman, 2024.

Domain	All participants (N = 150)			Patients (N = 85)			Attendees (N = 65)		
	Cases	Non-cases		Cases	Non-cases		Cases	Non-cases	
	M (SD)	M (SD)	P	M (SD)	M (SD)	P	M (SD)	M (SD)	P
Visual Processing Accuracy (RVPA)	0.78 (0.09)	0.85 (0.06)	0.01	0.80 (0.07)	0.86 (0.05)	0.001	0.74 (0.10)	0.84 (0.06)	0.01
Response Latency (RVPMDL)	638.48 (211.61)	539.76 (161.62)	0.01	619.39 (192.13)	535.00 (151.60)	0.04	668.48 (240.93)	544.36 (174.40)	0.04
Immediate Recall (PRMPCI)	74.69 (18.71)	81.19 (6.86)	0.03	72.73 (19.06)	82.59 (16.01)	0.01	77.78 (18.17)	79.55 (17.84)	0.71
Delayed Recall (PRMPCD)	62.45 (17.30)	70.89 (19.49)	0.01	62.96 (18.20)	73.18 (20.36)	0.02	61.64 (16.19)	68.31 (18.31)	0.16
Planning Efficiency (OTSPSFC)	6.76 (3.97)	9.83 (2.77)	0.01	7.03 (4.24)	9.92 (2.69)	0.01	6.33 (3.57)	9.72 (2.88)	0.01
Planning Latency (OTSMDLFC)	11129.70 (8,628.51)	12484.20 (5,935.00)	0.25	10951.15 (8904.45)	12815.58 (6004.42)	0.25	11441.03 (8384.38)	12092.57 (5896.67)	0.71
Spatial Working Memory Errors (All) (SWMBE468)	15.02 (9.47)	12.94 (9.59)	0.20	15.00 (10.14)	12.29 (9.71)	0.22	15.05 (8.57)	13.70 (9.51)	0.59
Strategy Use (SWMS)	8.59 (2.06)	8.21 (2.25)	0.30	8.48 (2.33)	8.34 (2.21)	0.78	8.76 (1.58)	8.05 (2.30)	0.20
Spatial Memory Errors (4 Boxes) (SWMBE4)	1.09 (1.55)	0.74 (1.22)	0.15	1.00 (1.46)	0.69 (1.20)	0.29	1.24 (1.70)	0.80 (1.27)	0.30
Spatial Memory Errors (6 Boxes) (SWMBE6)	3.87 (3.34)	3.06 (3.66)	0.18	3.91 (3.35)	2.92 (3.59)	0.21	3.81 (3.40)	3.23 (3.77)	0.55
Spatial Memory Errors (8 Boxes) (SWMBE8)	10.06 (6.39)	9.14 (7.01)	0.43	10.09 (7.13)	8.67 (7.37)	0.38	10.00 (5.19)	9.68 (6.59)	0.85

In subgroup analyses, patients with Hikikomori showed significantly poorer performance across multiple domains, including visual processing accuracy, memory recall, and planning efficiency. Among attendees, significant differences were observed for visual processing accuracy and planning efficiency; however, differences in immediate and delayed recall were not statistically significant. Supplementary nonparametric analyses (Mann–Whitney U tests) produced comparable results, supporting the robustness of these findings.

### Association between hikikomori status and cognitive performance

Table 4 presents the crude and adjusted associations between HiDE-I case status and cognitive performance for the full sample and the patient and attendee subgroups. In the full sample, case status was associated with lower visual-processing accuracy, slower response latency, poorer immediate and delayed recall, and lower planning efficiency after adjustment for sociodemographic variables. Among patients, adjusted associations remained statistically significant for immediate recall, delayed recall, and planning efficiency; the association with response latency was not statistically significant. Among attendees, adjusted associations were statistically significant for visual-processing accuracy and planning efficiency. Delayed recall was at the conventional significance boundary (p = .05), whereas immediate recall, response latency, planning latency, and the spatial working-memory measures were not statistically significant. These subgroup findings should be interpreted cautiously because of the smaller sample sizes and multiple outcomes examined.

**Table 4.** Crude and adjusted linear regression models of Hikikomori status and cognitive performance, Oman, 2024.

Cognitive domain	All participants (N = 150)		Patients (N = 85)		Attendees (N = 65)	
	Crude β (p)	Adjusted β (p)	Crude β (p)	Adjusted β (p)	Crude β (p)	Adjusted β (p)
Visual processing accuracy (RVPA)	−0.440 (0.010)	−0.453 (0.001)	−0.422 (0.010)	−0.408 (0.001)	−0.506 (0.001)	−0.585 ((0.001)
Response latency (RVPMDL)	0.255 (0.020)	0.266 (0.001)	0.238 (0.020)	0.203 (0.060)	0.285 (0.030)	0.361 (0.080)
Immediate recall (PRMPCI)	−0.176 (0.030)	−0.201 (0.019))	−0.271 (0.010)	−0.292 (0.001)	−0.047 (0.710)	−0.083 (0.600)
Delayed recall (PRMPCD)	−0.213 (0.010)	−0.255 (0.003)	−0.247 (0.020)	−0.261 (0.020)	−0.176 (0.160)	−0.275 (0.050)
Planning efficiency (OTSPSFC)	−0.416 (0.010)	−0.453 (0.001)	−0.389 (0.010)	−0.410 (0.001)	−0.460 (0.010)	−0.554 (0.001)
Planning latency (OTSMDLFC)	−0.093 (0.260)	−0.098 (0.251)	−0.126 (0.250)	−0.150 (0.200)	−0.048 (0.700)	−0.092 (0.490)
Spatial working-memory errors (SWMBE468)	0.105 (0.200)	0.142 (0.096)	0.134 (0.220)	0.142 (0.220)	0.069 (0.590)	0.169 (0.210)
Strategy use (SWMS)	0.085 (0.300)	0.085 (0.325)	0.030 (0.780)	0.026 (0.820)	0.160 (0.200)	0.179 (0.200)
Spatial-memory errors, 4 boxes (SWMBE4)	0.126 (0.130)	0.149 (0.079)	0.116 (0.290)	0.116 (0.290)	0.147 (0.240)	0.225 (0.100)
Spatial-memory errors, 6 boxes (SWMBE6)	0.109 (0.180)	0.123 (0.156)	0.138 (0.210)	0.083 (0.410)	0.075 (0.550)	0.159 (0.240)
Spatial-memory errors, 8 boxes (SWMBE8)	0.065 (0.430)	0.107 (0.210)	0.096 (0.380)	0.124 (0.290)	0.024 (0.850)	0.106 (0.430)

*Note:* Values are standardized β coefficients with p-values in parentheses. Adjusted models controlled for gender, age, education, income, employment status, governorate, and marital status.

Table 5 presents the standardized β coefficients for the association between HiDE-I case status and the cognitive outcomes in the adjusted full-sample models. The largest absolute standardized coefficients were observed for visual-processing accuracy and planning efficiency (β = −.453 for both), followed by response latency (β = .266), delayed recall (β = −.255), and immediate recall (β = −.201). All remaining cognitive domains showed non-significant associations. After Bonferroni correction, visual-processing accuracy (p = .001), planning efficiency (p = .001), response latency (p = .001), and delayed recall (p = .003) remained statistically significant. Immediate recall (unadjusted p = .019) did not meet the Bonferroni-adjusted threshold and should therefore be interpreted as suggestive. All five outcomes remained significant under the Benjamini–Hochberg false-discovery-rate procedure.

**Table 5.** Ranking of CANTAB cognitive outcomes by relative importance (standardized β).

Rank	Cognitive Domain	CANTAB Measure	Standardized β	p-value	Effect Direction
1	Visual Processing Accuracy	RVPA	−0.453	0.001	↓ Hikikomori
2	Planning Efficiency	OTSPSFC	−0.453	0.001	↓ Hikikomori
3	Delayed Recall	PRMPCD	−0.255	0.003	↓ Hikikomori
4	Immediate Recall	PRMPCI	−0.201	0.019	↓ Hikikomori
5	Response Latency	RVPMDL	+0.266	0.001	↑ (Slower in Hikikomori)
6	Planning Latency	OTSMDLFC	−0.098	0.25	↔ Non-significant
7	Spatial Working Memory Errors	SWMBE468	+0.142	0.09	↔ Non-significant
8	Strategy Use	SWMS	+0.085	0.32	↔ Non-significant
9	Spatial Memory Errors (4 boxes)	SWMBE4	+0.149	0.07	↔ Non-significant
10	Spatial Memory Errors (6 boxes)	SWMBE6	+0.123	0.15	↔ Non-significant
11	Spatial Memory Errors (8 boxes)	SWMBE8	+0.107	0.21	↔ Non-significant

(Values are standardized β coefficients from the adjusted regression model, ranked by absolute magnitude. Negative β reflects poorer cognitive performance among Hikikomori cases).

***Independence from Emotional Distress***

To evaluate whether the observed cognitive differences might reflect general psychiatric comorbidity rather than a Hikikomori-specific profile, we used DASS-21 depression, anxiety, and stress scores collected from all 150 CANTAB participants as part of the parent study. Hierarchical linear regression (sociodemographic covariates and patient/attendee status in Block 1, HiDE-I status in Block 2, DASS-21 subscales in Block 3; Table 6) showed that Hikikomori status contributed significant, substantial explained variance in every cognitive domain after adjustment (ΔR ^2^ = .037–.195, all p < .05), while emotional distress added negligible further variance once Hikikomori status was accounted for (ΔR ^2^ = .004–.012, all p > .50). Within HiDE-I-positive cases, Spearman correlations between depression severity and cognitive performance were non-significant for all five outcomes (n = 40 dual positive cases: p = .526–.920; n = 54 broader HiDE-I-positive group: p = .081–.889).

**Table 6.** Hierarchical linear regression examining the independent contribution of Hikikomori status and emotional distress (DASS-21) to CANTAB cognitive performance, after adjusting for sociodemographic factors and patient/attendee status (N = 150).

CANTAB domain	Block	Predictors added	R ^**2**^	ΔR ^**2**^	p
Visual Processing Accuracy (RVPA)	1	Demographics + patient status	.089	.089	.097
	2	HiDE-I status	.285	.195	<.001
	3	Emotional distress (DASS-21)	.297	.012	.507
Response Latency (RVPMDL)	1	Demographics + patient status	.084	.084	.125
	2	HiDE-I status	.150	.066	<.001
	3	Emotional distress (DASS-21)	.161	.011	.608
Immediate Recall (PRMPCI)	1	Demographics + patient status	.033	.033	.777
	2	HiDE-I status	.070	.037	<.001
	3	Emotional distress (DASS-21)	.074	.005	.881
Delayed Recall (PRMPCD)	1	Demographics + patient status	.048	.048	.524
	2	HiDE-I status	.110	.062	<.001
	3	Emotional distress (DASS-21)	.114	.004	.907
Planning Efficiency (OTSPSFC)	1	Demographics + patient status	.019	.019	.951
	2	HiDE-I status	.209	.191	<.001
	3	Emotional distress (DASS-21)	.221	.012	.565

Block 1 predictors: age, gender, education, income, employment status, governorate, marital status, patient-versus-attendee status. Block 2 predictor: HiDE-I Hikikomori status (0 = non-case, 1 = case). Block 3 predictors: DASS-21 depression, anxiety, and stress subscale scores. ΔR ^2^ = R ^2^ change contributed by each block.

To examine whether current emotional-symptom severity accounted for additional variance in cognitive performance, exploratory hierarchical linear regressions were conducted using DASS-21 depression, anxiety, and stress scores available for all 150 CANTAB participants. Sociodemographic variables and patient-versus-attendee status were entered in Block 1, HiDE-I case status in Block 2, and the three DASS-21 subscales in Block 3. HiDE-I status increased explained variance across the five principal cognitive outcomes (ΔR ^2^ = .037–.195), whereas the DASS-21 block added ΔR ^2^ = .004–.012 and was not statistically significant in any model (all p > .50).

Spearman correlations between depression severity and the five cognitive outcomes were also non-significant in the dual-positive group (n = 40) and the broader HiDE-I-positive group (n = 54). In these exploratory analyses, current DASS-21 symptom severity did not explain significant additional variance after HiDE-I status. This reduces, but does not eliminate, concern that current depression, anxiety, or stress symptoms account for the observed cognitive differences. DASS-21 is not a diagnostic interview, and diagnosis-specific variables, neurodevelopmental conditions, substance use, and medication exposure were not modeled; consequently, these results cannot establish a Hikikomori-specific cognitive profile.

Table 6 Hierarchical linear regression examining the independent contribution of Hikikomori status and emotional distress (DASS-21) to CANTAB cognitive performance, after adjusting for sociodemographic factors and patient/attendee status (N = 150). Block 1 predictors: age, gender, education, income, employment status, governorate, marital status, patient-versus-attendee status. Block 2 predictor: HiDE-I Hikikomori status (0 = non-case, 1 = case). Block 3 predictors: DASS-21 depression, anxiety, and stress subscale scores. ΔR ^2^ = R ^2^ change contributed by each block. In these exploratory models, the DASS-21 symptom block did not explain significant additional variance after HiDE-I status. This finding reduces, but does not eliminate, concern that current depression, anxiety, or stress symptom severity accounts for the observed differences. Because DASS-21 is not a diagnostic interview and medication and diagnosis-specific variables were not modeled, the results cannot establish a Hikikomori-specific cognitive profile.

## Discussion

The present study examined neurocognitive performance using cases identified through structured clinical assessment. Hikikomori has been extensively studied in East Asia, yet its cognitive aspects remain unexamined in the Middle East, leaving a significant literature gap. As cultural and societal factors influence its presentation, investigating Hikikomori in Oman is essential for developing region-specific diagnostic frameworks and interventions.

This study assesses cognitive performance in Hikikomori cases and non-cases, with a focus on executive function, memory recall, and processing speed. Given the scarcity of research in the region, it also examines socio-demographic characteristics, offering insights into Hikikomori within an Omani context. As the first study to examine cognitive functioning in Hikikomori individuals using CANTAB, this research provides novel insights into neurocognitive risk factors while addressing a critical gap in Middle Eastern literature.

### Executive function and processing speed

Hikikomori cases showed lower planning efficiency and slower response latency than non-cases after adjustment for sociodemographic variables. These findings indicate differences in selected aspects of executive and processing-speed performance associated with HiDE-I case status in this sample. Similar cognitive differences have been documented in several psychiatric conditions that may co-occur with Hikikomori ( Bourke et al., 2012; Gau & Shang, 2010). However, the present study did not determine whether psychiatric comorbidity accounted for these domain-specific findings. The observed cognitive differences may be associated with functional withdrawal, but the direction and mechanism of this association were not tested in this cross-sectional study.

Prior research indicates that deficits in response inhibition and attentional shifting, commonly seen in social anxiety disorder, may contribute to social withdrawal ( Pupi et al., 2025; Rhodes et al., 2005). Given the high prevalence of social anxiety in Hikikomori ( Kato et al., 2024), these cognitive difficulties are consistent with, and may plausibly contribute to, the maintenance of avoidance behaviors ( Lo et al., 2023), though the cross-sectional design cannot establish directionality. In addition to deficits in planning efficiency, Hikikomori cases also exhibited slower response times, suggesting broader differences in processing speed and cognitive efficiency that may further exacerbate withdrawal tendencies.

In this study, planning efficiency and visual processing accuracy showed the strongest associations with Hikikomori, which may reflect difficulties in goal-directed behavior and in the interpretation of environmental cues necessary for social interaction. These findings are consistent with evidence showing that executive dysfunction and attentional processing difficulties are linked to reduced social functioning and withdrawal in clinical populations ( Bourke et al., 2012; Kim et al., 2014; Luciana & Nelson, 2002). The minimal associations observed for spatial working memory and strategy use suggest that Hikikomori is characterized by selective rather than global cognitive impairment, a pattern also reported in recent neuropsychological research on Hikikomori ( Santona et al., 2023) and in studies demonstrating that only specific executive domains may be affected rather than overall cognitive capacity ( Rhodes et al., 2005). This selective vulnerability highlights the importance of interventions that target planning, cognitive flexibility, and visual–attentional processing, rather than assuming a broad cognitive decline among Hikikomori individuals.

### Memory recall deficits

Hikikomori cases performed less well than non-cases on immediate and delayed memory-recall measures in the unadjusted analyses. Both associations remained statistically significant in the primary adjusted models; however, immediate recall did not survive the Bonferroni correction and should be interpreted as suggestive. Memory differences have also been reported in social anxiety disorder and other psychiatric conditions ( Airaksinen et al., 2005). The present findings demonstrate an association between HiDE-I case status and memory performance but do not establish whether memory differences preceded, followed, or contributed to social withdrawal.

### Visual processing and attention

Hikikomori cases showed lower visual-processing accuracy than non-cases after adjustment for sociodemographic variables. This association remained statistically significant after correction for multiple testing. No statistically significant case–non-case differences were detected for spatial working-memory errors, planning latency, or strategy use. These non-significant results should not be interpreted as evidence that these domains were intact or equivalent between groups, because the study may have had limited precision for detecting smaller effects. These findings align with research showing that psychiatric conditions often affect distinct cognitive domains ( Bourke et al., 2012; Kim et al., 2014; Luciana & Nelson, 2002).

Given that social anxiety disorder (SAD) is one of the most common comorbidities in Hikikomori ( Lin et al., 2022; Pupi et al., 2025; Tateno et al., 2012), attentional and cognitive flexibility impairments—key features of SAD—are consistent with, and may plausibly contribute to, persistent withdrawal behaviors, though the cross-sectional design cannot establish directionality. SAD has been linked to difficulties in processing social cues and regulating attention and emotion ( Bourke et al., 2012), which provides a possible contextual interpretation of the observed findings; however, this mechanism was not directly tested in the present study.

Furthermore, neuroimaging studies have shown that chronic social withdrawal is associated with changes in brain regions responsible for visual processing and attention, including the hippocampus, ventral striatum, and prefrontal cortex ( Zovetti et al., 2021). These findings align with recent work on COVID-19-related isolation, which underscores the broader neurological and psychological consequences of prolonged social disengagement ( Roza et al., 2020).

### Spatial working memory and strategy use

Hikikomori cases showed lower performance in selected measures of planning, visual-processing accuracy, response latency, and memory recall. No statistically significant differences were detected in the spatial working-memory measures, planning latency, or strategy use. This pattern is consistent with domain-specific rather than uniformly lower cognitive performance; however, the non-significant findings do not demonstrate equivalence or preserved functioning. Larger studies are needed to determine whether smaller differences are present in these domains.

### Gender-based differences

Gender was included as a covariate in the adjusted regression models but was not formally tested as a moderator of the association between Hikikomori status and cognitive performance (i.e., no gender-by-case interaction term or gender-stratified model was fitted). The adjusted associations reported above therefore already account for gender differences in the sample but cannot speak to whether the Hikikomori–cognition association itself differs by gender. Testing this formally, ideally in a larger sample, is a priority for future research given the well-documented gender differences in cognitive performance in the general population ( Camarata & Woodcock, 2006; Herlitz & Rehnman, 2008).

### Socioeconomic factors and cognitive performance

Hikikomori cases in this sample were more likely to be unemployed and to report lower household income than non-cases, consistent with prior work linking prolonged social withdrawal to reduced workforce participation ( Nonaka & Sakai, 2021). Cases and non-cases differed descriptively in their employment and household-income distributions ( Table 2). Employment and income were included as covariates in the adjusted cognitive models; however, no interaction, mediation, or socioeconomic-stratified analysis was performed. The present study therefore cannot determine whether socioeconomic factors caused social withdrawal or modified the association between HiDE-I case status and cognitive performance. These findings should be interpreted as characteristics of the study sample rather than evidence of a causal socioeconomic mechanism.

### Implications for intervention

The cognitive differences observed in this study may identify domains worth assessing when planning future interventions. However, the present study did not evaluate cognitive remediation or any clinical intervention and therefore cannot establish treatment effectiveness. Future intervention studies should determine whether addressing planning, attention, processing speed, or memory improves functional or social outcomes among people with Hikikomori.

### Limitations

Several limitations should be considered. First, the case–control design precludes causal inference; cognitive differences may precede social withdrawal or emerge because of prolonged disengagement. Longitudinal studies are needed to clarify directionality. Second, multiple cognitive domains were examined, raising the possibility of type I error due to multiple comparisons. Although four of the five principal findings remained statistically significant after Bonferroni correction, immediate recall did not survive this conservative adjustment. The immediate-recall finding should therefore be interpreted cautiously and requires replication. Third, participants were recruited from a hospital-based behavioral medicine clinic, introducing potential selection bias that likely biases the sample toward more severe, help-seeking presentations and would tend to overestimate cognitive impairment relative to a community-representative sample; while inclusion of non-patient attendees broadened representation and may partly offset this, results may not fully generalize to community samples. Fourth, formal psychiatric diagnoses among non-case patients were not systematically catalogued. However, a post-hoc analysis using DASS-21 scores collected from the full CANTAB sample (see Independence from Emotional Distress, above) found that depression, anxiety, and stress severity did not explain the observed cognitive differences, and a within-patient subgroup analysis ( Table 4), in which both cases and non-cases were drawn from the psychiatric patient population, also showed persisting cognitive differences by Hikikomori status. Together these reduce, though do not eliminate, the concern that general psychiatric comorbidity rather than Hikikomori-specific factors drives the findings. Fifth, CANTAB has not been formally validated against Omani normative data; it was selected for its non-verbal, examiner-independent design and its use across diverse clinical and non-clinical populations internationally ( Bourke et al., 2012; Green et al., 2019; Kim et al., 2014), which supports cross-cultural applicability. Although the computerized, largely non-verbal format reduces some language and examiner effects, it does not eliminate cultural, educational, or contextual influences. Local psychometric and normative studies are therefore needed. Finally, medication status was not systematically assessed; psychotropic medications may influence attention, processing speed, and memory in either direction, and this — along with comorbidities beyond depression/anxiety/stress (e.g., ADHD, substance use) — remains an unaddressed potential confound for future replication.

Future research should combine longitudinal cognitive assessment with structured measurement of psychiatric diagnoses, neurodevelopmental conditions, emotional symptoms, and medication exposure. Structural and functional neuroimaging may help determine whether the observed behavioral differences correspond to changes in neural systems supporting attention, planning, and memory ( Zovetti et al., 2021). Longitudinal designs would be required to examine whether these cognitive and neural differences precede prolonged withdrawal, emerge following withdrawal, or change during recovery or intervention. Cross-sectional neuroimaging alone would not establish a Hikikomori-specific mechanism. Because exposure and outcome were measured cross-sectionally, the study cannot determine whether cognitive differences preceded withdrawal, followed it, or arose from shared psychiatric, developmental, medication-related, or social factors.

## Conclusion

This study found that Hikikomori in Oman is associated with selective neurocognitive differences, particularly in planning efficiency, sustained attentional accuracy, processing speed, and visual memory recall. These findings extend existing Hikikomori research into a Middle Eastern context and suggest that cognitive inefficiencies may accompany prolonged social withdrawal. Because the design was cross-sectional and diagnosis-specific, neurodevelopmental, substance-related, and medication variables were not modeled, these results describe an association and cannot establish a Hikikomori-specific cognitive profile.

Rather than indicating global cognitive decline, the results point to specific domains that may contribute to functional disengagement. Future longitudinal and multimodal studies are needed to clarify causality, disentangle comorbidity effects, and determine whether these cognitive patterns change with recovery or intervention.

## Ethics approval and consent to participate

Ethical approval was obtained from the Sultan Qaboos University Hospital Ethics Committee (MREC #2260). Written informed consent was obtained from all participants.

## Availability of data and materials

The datasets generated and/or analyzed during the current study are not publicly available due to ethical and confidentiality restrictions imposed by the Sultan Qaboos University Hospital Ethics Committee (MREC #2260), as the data contain sensitive clinical and potentially identifiable participant information.

The Ethics Committee does not permit open public sharing of individual-level clinical data; however, data may be made available upon reasonable request for academic and research purposes.

Researchers who wish to access the data must submit a formal request outlining the purpose of use and intended analyses. Requests are subject to approval by the corresponding author and, where applicable, the Sultan Qaboos University Hospital Ethics Committee.

Requests for data access can be directed to:

Professor Yahya M. Al-Farsi (Corresponding Author).

Email: ymfarsi@squ.edu.om

Access will be granted under conditions that ensure compliance with ethical guidelines, data protection regulations, and participant confidentiality.

## Authors’ information

NAK is a PhD candidate in Public Health and Epidemiology in Sultan Qaboos University, Oman.
