# Supplementary material for: Executive Dysfunction and Memory Impairments in Hikikomori: A Neuropsychological Case-Control Study from Oman
Source: F1000Res. 2026 Sep 22;15:874. Originally published 2026 Jun 5. [Version 4] doi: 10.12688/f1000research.181828.4 (PMC13679784; doi:10.12688/f1000research.181828.4)
Supplement: Peer review history

# Reviewer report: Reviewer response for version 4

**DOI:** 10.5256/f1000research.210781.r523435

**Published:** 2026-10-05

**Authors:** Helena Vélez-Botero

**Affiliations:** 1 National University of Colombia, Bogotá, Colombia

Thank the authors for their careful and constructive response to my previous report. The two outstanding concerns have been satisfactorily addressed.

 The Abstract and Conclusion now use language consistent with the caution expressed in the Discussion. The classification rules for the "at-risk" and "resembling" HiDE-I categories are now specified, so the case definition is reproducible by other researchers. 

 I appreciate author dedication. Together with the revisions made earlier, the manuscript now presents its findings in a clear, balanced, and thoughtful manner, with appropriate nuance and careful consideration of the evidence.

 I have no further concerns and recommend approval.

Is the work clearly and accurately presented and does it cite the current literature?

Partly

If applicable, is the statistical analysis and its interpretation appropriate?

Partly

Are all the source data underlying the results available to ensure full reproducibility?

Yes

Is the study design appropriate and is the work technically sound?

Partly

Are the conclusions drawn adequately supported by the results?

Partly

Are sufficient details of methods and analysis provided to allow replication by others?

No

Reviewer Expertise:

Health psychology

I confirm that I have read this submission and believe that I have an appropriate level of expertise to confirm that it is of an acceptable scientific standard.

# Reviewer report: Reviewer response for version 4

**DOI:** 10.5256/f1000research.210781.r523434

**Published:** 2026-09-25

**Authors:** Alicia Nevriana

**Affiliations:** 1 Karolinska Institutet, Stockholm, Sweden

Thank you for incorporating my comments in this version. I have no further comments to make.

Is the work clearly and accurately presented and does it cite the current literature?

Partly

If applicable, is the statistical analysis and its interpretation appropriate?

Partly

Are all the source data underlying the results available to ensure full reproducibility?

Partly

Is the study design appropriate and is the work technically sound?

Yes

Are the conclusions drawn adequately supported by the results?

Partly

Are sufficient details of methods and analysis provided to allow replication by others?

No

Reviewer Expertise:

Epidemiology

I confirm that I have read this submission and believe that I have an appropriate level of expertise to confirm that it is of an acceptable scientific standard.

# Reviewer report: Reviewer response for version 3

**DOI:** 10.5256/f1000research.209425.r517287

**Published:** 2026-09-15

**Authors:** Helena Vélez-Botero, Andrés Camilo Delgado Reyes

**Affiliations:** 1 Universidad de Manizales (Ringgold ID: 27992), Manizales, Caldas, Colombia; 2 National University of Colombia, Bogotá, Colombia

This new revision represents a substantial and largely successful response to reviewer concerns: the authors conducted a genuine reference audit, added appropriate multiple-comparison corrections, tempered unsupported causal claims about gender and socioeconomic factors, and transparently reported an exploratory DASS-21 analysis. However, two issues remain insufficiently addressed: first, the Abstract and Conclusion still assert unqualified deficits (*"Hikikomori individuals in Oman exhibit significant deficits in executive function..."*) despite the Discussion's explicit admission that *"these results cannot establish a Hikikomori-specific cognitive profile"* — this inconsistency should be resolved by aligning all sections to the same epistemic caution; second, the HiDE-I scoring algorithm for the "at-risk" and "resembling" subcategories remains underspecified ( *"according to the administered HiDE-I criteria"* merely cites an external source rather than detailing the classification rule), which still limits full replicability.

Is the work clearly and accurately presented and does it cite the current literature?

Partly

If applicable, is the statistical analysis and its interpretation appropriate?

Partly

Are all the source data underlying the results available to ensure full reproducibility?

Yes

Is the study design appropriate and is the work technically sound?

Partly

Are the conclusions drawn adequately supported by the results?

Partly

Are sufficient details of methods and analysis provided to allow replication by others?

No

Reviewer Expertise:

Health psychology

We confirm that we have read this submission and believe that we have an appropriate level of expertise to confirm that it is of an acceptable scientific standard, however we have significant reservations, as outlined above.

# Response

**Published:** 2026-09-15

**Authors:** Nutaila Al Kharusi

**Affiliations:** Family Medicine Public Health, Sultan Qaboos University College of Medicine and Health Science, Muscat, Muscat Governorate, Oman

Dear Reviewer, 

 Thank you for your careful reading of our revised manuscript and for your positive comments on the changes we made in version 3. We have now addressed the two remaining points.

**1. Abstract and Conclusion not consistent with the Discussion**

 You are right. The Abstract still said that Hikikomori cases "exhibit significant deficits", while the Discussion clearly says the results cannot establish a Hikikomori-specific cognitive profile. We have corrected this.

 In the Abstract, we changed "significant impairments" in the Results to "significantly poorer performance", and we rewrote the Conclusion. It now describes the findings as an association between HiDE-I case status and poorer cognitive performance in a cross-sectional sample, and it states clearly that psychiatric diagnoses, neurodevelopmental conditions, substance use and medication were not modelled, so the results cannot establish a Hikikomori-specific cognitive profile or its direction.

 In the main Conclusion, we changed "This study demonstrates" to "This study found" and added a sentence with the same caution about the cross-sectional design and the unmodelled variables. All three sections now use the same level of caution.

**2. HiDE-I classification rule for the at-risk and resembling subcategories**

 We agree that citing Teo et al. was not enough. We have now written the full HiDE-I rule in the Methods (Case ascertainment and selection of cases). The HiDE-I uses three criteria in order: Criterion I, physical withdrawal (three days or fewer per week with at least one hour of activity outside the home during the past month); Criterion II, distress or impairment linked to the withdrawal (distress or loneliness, family concern, or impairment in work, study, home life or friendships); and Criterion III, duration of withdrawal.

 Participants who did not meet Criterion I were non-cases. Participants who met Criterion I but not Criterion II were classified as Hikikomori resemblance. Participants who met Criteria I and II were classified by duration: pathological Hikikomori if six months or more, and at-risk Hikikomori if less than six months. In the HiDE algorithm the under-six-month group is divided into at-risk (under three months) and pre-pathological (three to six months); in our study these two were combined into one at-risk category. So we used four groups: non-case, resemblance, at-risk and pathological. All participants in the three positive groups were counted as HiDE-I-positive cases, regardless of their HQ-25 score.

 The full rule is now in the manuscript itself, so another researcher can apply the same classification.

 We thank the reviewer again for the helpful comments.

# Reviewer report: Reviewer response for version 2

**DOI:** 10.5256/f1000research.203333.r501736

**Published:** 2026-08-03

**Authors:** Helena Vélez-Botero, Andrés Camilo Delgado Reyes

**Affiliations:** 1 National University of Colombia, Bogotá, Colombia; 2 Universidad de Manizales (Ringgold ID: 27992), Manizales, Caldas, Colombia

This manuscript addresses an important and clinically relevant question about social withdrawal by exploring the neurocognitive profile associated with Hikikomori using standardized cognitive assessments. The integration of neuropsychological measures with psychiatric evaluation represents a valuable contribution to a field where objective cognitive evidence remains relatively limited.

 The abstract is accurate and consistent with the body of the manuscript. The Introduction provides a solid theoretical foundation. However, some statements appear to attribute findings to references that may not represent the original source of the reported data, while others cite secondary, methodological, or review publications as direct evidence for empirical associations. The authors should ensure that all references accurately support the corresponding statements and clearly distinguish primary evidence from secondary or methodological sources where appropriate.

 The overall study design is conceptually sound. However, the non-case group combines (a) psychiatric patients without Hikikomori and (b) healthy companions with no psychiatric diagnosis. Because the literature cited by the authors themselves indicates that depression and anxiety are associated with cognitive deficits similar to those reported here (e.g., processing speed, memory, and planning), and because psychiatric comorbidity was neither measured nor statistically adjusted for, it cannot be ruled out that the observed differences reflect general psychiatric comorbidity rather than a Hikikomori-specific cognitive profile. This represents a direct threat to the validity of the study's central conclusion.

 I also agree with the previous reviewer that several essential methodological details required for replication are missing:

- No description of the HiDE-I item content or the scoring system used to distinguish the three case subcategories.
- No information on whether the CANTAB battery has been validated for this population (e.g., normative data or psychometric properties).
- No detailed description of the sociodemographic questionnaire (e.g., exact items, income categories, employment classifications).
- No explanation of the convenience sampling strategy used to recruit participants.
- The participant flow diagram does not explain why only 454 of the 1,200 screened individuals proceeded to the diagnostic interview.

The Results section demonstrates good statistical transparency and an appropriate application of statistical methods. The primary analyses are correctly conducted and clearly described. However, several methodological issues should be addressed:

- Eleven cognitive domains were analyzed across multiple regression models without adjustment for multiple comparisons (e.g., Bonferroni correction or false discovery rate).
- Sample size was calculated using Rothman's Episheet for case-control odds ratios, whereas the primary analyses were based on linear regression models.
- The discrepancy between the assumed (1:1) and achieved (54:96; approximately 1:1.8) case-control ratio is not discussed in terms of its potential impact on statistical power.
- Several interpretative statements are not directly supported by the analyses. For example, the Discussion concludes that "social withdrawal may affect both genders similarly" and extensively speculates about socioeconomic factors "contributing to" social withdrawal, despite the absence of sex-by-case interaction analyses or socioeconomic-stratified models. These interpretations extend beyond the evidence presented.

The Discussion is well organized by cognitive domain. The principal quantitative findings are adequately supported by the adjusted analyses and are interpreted using generally appropriate and cautious language. Nevertheless, several interpretative statements exceed what the results can support:

- Attributing the observed cognitive deficits specifically to Hikikomori despite the absence of control for psychiatric comorbidity.
- Drawing conclusions regarding gender differences and socioeconomic influences without corresponding formal statistical analyses.
- Using expressions such as "may reinforce avoidance behaviors" and similar statements that, although theoretically plausible and supported by the cited literature, are occasionally presented with greater certainty than is warranted by a cross-sectional case-control design.

Is the work clearly and accurately presented and does it cite the current literature?

Partly

If applicable, is the statistical analysis and its interpretation appropriate?

Partly

Are all the source data underlying the results available to ensure full reproducibility?

Yes

Is the study design appropriate and is the work technically sound?

Partly

Are the conclusions drawn adequately supported by the results?

Partly

Are sufficient details of methods and analysis provided to allow replication by others?

No

Reviewer Expertise:

Health psychology

We confirm that we have read this submission and believe that we have an appropriate level of expertise to confirm that it is of an acceptable scientific standard, however we have significant reservations, as outlined above.

# Response

**Published:** 2026-08-26

**Authors:** Nutaila Al Kharusi

**Affiliations:** Family Medicine Public Health, Sultan Qaboos University College of Medicine and Health Science, Muscat, Muscat Governorate, Oman

We sincerely thank Dr Vélez-Botero and Dr Delgado Reyes for their careful, constructive, and detailed evaluation of our manuscript. We appreciate their recognition of the clinical relevance of the research question and the potential contribution of standardized neuropsychological assessment to the Hikikomori literature.

 In response to their concerns, we have substantially revised the manuscript. The principal changes include:

1. Auditing the references and replacing secondary, bibliometric, and methodological sources with appropriate primary evidence.
2. Clarifying the composition of the HiDE-I-negative non-case group and tempering claims of a Hikikomori-specific cognitive profile.
3. Adding an exploratory analysis using DASS-21 depression, anxiety, and stress scores, while acknowledging that these symptom measures do not replace structured psychiatric diagnoses or medication data.
4. Expanding the description of the HiDE-I, the study-specific case definition, the CANTAB assessment, and the sociodemographic questionnaire.
5. Explaining the convenience-sampling procedure and why 454 of the 1,200 screened participants completed the HiDE-I.
6. Applying sensitivity analyses for multiple comparisons and interpreting immediate recall cautiously because it did not survive Bonferroni correction.
7. Clarifying that the original sample-size calculation belonged to the broader parent study and was not specifically designed for the continuous CANTAB outcomes.
8. Explaining the difference between the planned and achieved case-to-non-case ratios without claiming that the unequal allocation increased power.
9. Removing unsupported conclusions concerning gender and socioeconomic effects.
10. Revising causal and mechanistic language throughout the Discussion and Conclusion to reflect the limitations of the cross-sectional design.

Our detailed point-by-point responses are provided below.

 Comment 1: Accuracy and appropriateness of the references

 The reviewers noted that some statements relied on secondary, bibliometric, methodological, or review publications rather than the primary sources of the reported findings.

**Response:**

 Thank you. We agree and conducted a claim-by-claim audit of the references used in the Introduction and Discussion. We removed bibliometric and protocol publications where they had been presented as direct empirical evidence. In particular, Cai et al. (2023), a bibliometric publication, and Pozza et al. (2019), a systematic-review protocol, are no longer cited as primary evidence of psychiatric comorbidity.

 We also replaced the secondary attribution of the 54.5% lifetime-comorbidity estimate with the primary source, Koyama et al. (2010), and separated evidence concerning psychiatric comorbidity in Hikikomori from evidence concerning cognitive performance in depression and anxiety.

 The revised text reads:

 “Studies of Hikikomori have reported co-occurring mood, anxiety, and other psychiatric disorders, although estimates vary according to the population, case definition, and assessment method (Koyama et al., 2010; Teo et al., 2020). In a Japanese community survey of adults aged 20–49 years, 54.5% of respondents with a lifetime history of Hikikomori had also experienced a lifetime mood, anxiety, impulse-control, or substance-related disorder (Koyama et al., 2010).”

 Comment 2: Heterogeneity of the non-case group and psychiatric comorbidity

 The reviewers noted that the non-case group combined psychiatric patients without Hikikomori and non-patient companions and that the observed cognitive differences might reflect general psychiatric comorbidity rather than a Hikikomori-specific profile.

**Response:**

 We agree that the composition of the non-case group and the absence of diagnosis- and medication-adjusted primary models limit the extent to which the observed cognitive differences can be attributed specifically to Hikikomori. We have therefore replaced “healthy controls” with “HiDE-I-negative non-cases” throughout the manuscript and have tempered the central conclusion.

 All 150 participants in the CANTAB sample also completed the DASS-21 as part of the parent study. This allowed us to conduct an exploratory post-hoc hierarchical analysis in which sociodemographic variables and patient-versus-attendee status were entered first, HiDE-I case status was entered second, and continuous DASS-21 depression, anxiety, and stress scores were entered third. The addition of the DASS-21 symptom block explained little additional variance after HiDE-I status. We also examined correlations between depression severity and the five principal cognitive outcomes within HiDE-I-positive participants; none reached statistical significance.

 However, we recognize that DASS-21 measures current emotional symptoms and is not a structured diagnostic interview. It does not control for diagnosis type, neurodevelopmental conditions, substance use, medication exposure, or other potentially relevant confounders. Furthermore, the additional models were exploratory and included numerous predictors relative to the sample size. We therefore do not interpret these analyses as establishing a Hikikomori-specific cognitive profile.

 The revised Results text reads:

 “In an exploratory post-hoc analysis of the full CANTAB sample (N=150), hierarchical linear models entered sociodemographic variables and patient-versus-attendee status in Block 1, HiDE-I case status in Block 2, and continuous DASS-21 depression, anxiety, and stress scores in Block 3. HiDE-I status increased the explained variance across the five principal cognitive outcomes (ΔR²=.037–.195), whereas the DASS-21 symptom block added ΔR²=.004–.012. Exact model statistics are reported in Table 6. Because this analysis was post-hoc, included numerous predictors relative to the sample size, and measured emotional symptoms rather than psychiatric diagnoses, it reduces but does not eliminate concern about psychiatric confounding.”

 The revised Discussion states:

 “The findings demonstrate cognitive differences associated with HiDE-I case status in this sample; they do not establish a Hikikomori-specific neurocognitive profile. Residual confounding by psychiatric diagnosis, neurodevelopmental conditions, medication exposure, substance use, and other unmeasured factors remains possible.”

 We have also clarified that the patient-only subgroup comparison controls for recruitment source and patient status but does not hold psychiatric diagnosis, illness severity, or medication exposure constant.

 Comment 3: HiDE-I item content and case classification

 No description was provided of the HiDE-I questions or the system used to distinguish the three case subcategories.

**Response:**

 We agree and have added a concise description of the interview domains and the exact three-category case definition used in the neurocognitive study.

 The revised text reads:

 “The HiDE-I is a structured clinician-administered interview assessing the frequency and purpose of outings, duration of withdrawal, social participation and interaction, and distress, concern by others, or functional impairment associated with withdrawal. For this neurocognitive study, participants classified as pathological, at-risk, or resembling Hikikomori were included as HiDE-I-positive cases, irrespective of HQ-25 score; participants meeting none of these categories were classified as non-cases. Pathological Hikikomori involved withdrawal lasting at least six months with significant distress or functional impairment. At-risk and resembling classifications captured emerging or subthreshold withdrawal presentations according to the administered HiDE-I criteria.”

 We use “determine” or “classify case status” rather than “confirm case status” because the case group included pathological, at-risk, and resembling presentations rather than only confirmed pathological cases.

 Comment 4: CANTAB validation in the Omani population

 No information was provided about whether CANTAB had been validated in this population.

**Response:**

 Thank you. Omani population-specific normative data and formal local validation were not available for the present study. We have stated this explicitly and added it as a limitation. CANTAB was selected because of its standardized computerized administration, automatic response recording, and relatively limited verbal demands. However, these characteristics do not eliminate cultural, educational, or contextual influences.

 The revised limitation reads:

 “Omani population-specific normative data and formal local validation for this CANTAB battery were not available to this study. Although the computerized, largely non-verbal format reduces some language and examiner effects, it does not eliminate cultural, educational, or contextual influences. Local psychometric and normative studies are therefore needed.”

 Comment 5: Description of the sociodemographic questionnaire

 The reviewers requested the questionnaire items and the categories used for income, employment, and other characteristics.

**Response:**

 We agree and have expanded the Methods to describe the variables collected and the analytical categories used. We have also aligned the Methods categories with those displayed in Table 2.

 The revised text reads:

 “Participants completed a structured self-report sociodemographic questionnaire covering age, gender, marital status, education, monthly household income, employment status, governorate of residence, and whether they had children. For the present analysis, age was grouped as 18–29, 30–39, and ≥40 years; income as <1,000, 1,000–1,999, 2,000–2,999, and ≥3,000 OMR; employment as employed or unemployed; marital status as never married or ever married; and education as <12, 12, or >12 years. Governorates represented in the sample are listed in Table 2.”

 Comment 6: Convenience sampling strategy

 No explanation was provided of how the convenience sample was recruited.

**Response:**

 We agree and have clarified that the CANTAB group was a non-random convenience subsample of participants who completed the HiDE-I. We have limited the description to the recruitment procedures documented in the study records.

 The revised text reads:

 “Participants who completed the HiDE-I and met eligibility criteria for the CANTAB sub-study were invited to complete neuropsychological testing. Selection was non-random and depended on participant consent and logistical feasibility within the clinical setting. The resulting CANTAB group should therefore be regarded as a convenience subsample rather than a representative random sample of all 454 interviewed participants.”

 We have also added this sampling process to the Limitations because it may affect the representativeness and generalizability of the neurocognitive sample.

 Comment 7: Why only 454 of the 1,200 screened participants completed the HiDE-I

 The participant-flow diagram did not explain why only 454 participants proceeded to the diagnostic interview.

**Response:**

 Thank you. We have clarified that the difference was primarily related to the protocol timeline. The HiDE-I was incorporated into the study on 1 January 2024; participants recruited before that date completed HQ-25 screening only. From 1 January 2024 onward, all eligible participants were invited to complete the HiDE-I irrespective of their HQ-25 score. Completion also depended on consent, interviewer availability, and logistical feasibility.

 The revised Methods and Figure 1 note read:

 “The HiDE-I was incorporated into the protocol from 1 January 2024; participants recruited earlier completed HQ-25 screening only. From that date, all eligible participants were invited to complete the HiDE-I irrespective of HQ-25 score. A total of 454 completed the interview; completion depended on consent, interviewer availability, and logistical feasibility and was not determined by HQ-25 positivity.”

 Comment 8: Multiple comparisons

 Eleven cognitive outcomes were analyzed without correction for multiple testing.

**Response:**

 Thank you. No multiplicity correction was included in the previous version. We have now applied multiplicity sensitivity analyses to the 11 adjusted case-status tests.

 The Statistical Analysis section now states:

 “Because 11 cognitive outcomes were examined, multiplicity was assessed using a Bonferroni family-wise significance threshold of α=.0045 (.05/11). A Benjamini–Hochberg false-discovery-rate correction (q=.05) was also conducted as a sensitivity analysis.”

 The revised Results state:

 “After Bonferroni correction, the associations with visual-processing accuracy (p=.001), planning efficiency (p=.001), response latency (p=.001), and delayed recall (p=.003) remained statistically significant. Immediate recall (unadjusted p=.019) did not meet the Bonferroni-adjusted threshold and should therefore be interpreted as suggestive. All five outcomes remained significant under the Benjamini–Hochberg false-discovery-rate procedure.”

 The Abstract, Discussion, and Limitations have also been revised so that immediate recall is not presented as a confirmatory finding after the conservative Bonferroni correction.

 Comment 9: Sample-size calculation

 The sample-size calculation was based on case–control odds ratios, whereas the primary analyses used continuous outcomes and linear regression.

**Response:**

 We agree. The Episheet calculation was developed for the broader parent case–control study and was not specifically tailored to the continuous CANTAB outcomes examined in this neurocognitive sub-study. We have clarified this distinction and removed post-hoc power statements based on the observed effect sizes.

 The revised text reads:

 “Sample size for the broader parent case–control study was calculated a priori using Rothman’s Episheet and was based on odds-ratio detection, assuming a control-to-case ratio of 1:1, a Zα value of 1.96, an exposure prevalence of 0.15, and 80% power to detect an odds ratio of 2.0. This calculation was not specifically tailored to the continuous CANTAB outcomes examined in the present neurocognitive sub-study. A two-group calculation based on an anticipated mean difference and variance would have been more directly appropriate.”

 We also acknowledge that the study may have had limited precision for detecting small effects and that non-significant findings should not be interpreted as evidence of equivalence.

 Comment 10: Difference between the planned and achieved case-to-non-case ratio

 The assumed ratio was 1:1, whereas the achieved ratio was 54:96, or approximately 1:1.8.

**Response:**

 Thank you. We have clarified the achieved allocation without claiming that it was more efficient than equal allocation.

 The revised text reads:

 “The achieved case-to-non-case ratio was 54:96 (approximately 1:1.8), rather than the 1:1 ratio assumed in the parent-study calculation. With a fixed total sample size and similar group variances, unequal allocation generally provides less precision than equal allocation; however, recruiting additional non-cases can improve precision when the number of available cases is constrained. Because the original calculation was not based on the continuous CANTAB outcomes, the power implications of the achieved ratio were not quantified.”

 Comment 11: Unsupported gender and socioeconomic interpretations

 The reviewers noted that conclusions about gender similarity and socioeconomic contributions were not supported by interaction or stratified analyses.

**Response:**

 We agree. Gender, employment, and income were included as covariates, but no gender-by-case interaction, socioeconomic interaction, mediation analysis, or stratified model was conducted. We have removed the unsupported conclusion that social withdrawal affects both genders similarly and have eliminated language implying that socioeconomic characteristics caused or modified the observed cognitive differences.

 The revised gender text reads:

 “Gender was included as a covariate in the adjusted regression models, but no case-status-by-gender interaction or gender-stratified analysis was performed. The present results therefore cannot determine whether the association between HiDE-I case status and cognitive performance differs by gender. Future studies with larger samples should formally examine gender as a potential moderator of this association.”

 The revised socioeconomic text reads:

 “Cases and non-cases differed descriptively in their employment and household-income distributions (Table 2). Employment and income were included as covariates in the adjusted cognitive models; however, no interaction, mediation, or socioeconomic-stratified analysis was performed. The present study therefore cannot determine whether socioeconomic factors caused social withdrawal or modified the association between HiDE-I case status and cognitive performance. These findings should be interpreted as characteristics of the study sample rather than evidence of a causal socioeconomic mechanism.”

 Comment 12: Causal and mechanistic interpretation

 The reviewers noted that expressions such as “may reinforce avoidance behaviors” were sometimes presented with greater certainty than warranted by the cross-sectional design.

**Response:**

 We agree and have revised causal and mechanistic statements throughout the Abstract, Discussion, and Conclusion. Cognitive findings are now described as differences associated with HiDE-I case status rather than as effects caused by Hikikomori. We have also removed language suggesting that non-significant findings demonstrate intact functioning and have clarified that the proposed links with avoidance and functional disengagement were not directly tested.

 Examples of the revisions include:

- “Hikikomori-specific deficits” was changed to “cognitive differences associated with HiDE-I case status.”
- “Specific vulnerability” was changed to “lower performance observed in this sample.”
- “Spatial working memory remained intact” was changed to “no statistically significant difference was detected in the spatial working-memory measures.”
- “May reinforce or exacerbate withdrawal” was changed to “may be associated with withdrawal; this pathway was not tested.”
- “Neurocognitive risk factors” was changed to “neurocognitive correlates.”

We also added the following statement:

 “Because exposure and outcome were measured cross-sectionally, the present study cannot determine whether cognitive differences preceded withdrawal, followed it, or arose from shared psychiatric, developmental, medication-related, or social factors.”

 We thank the reviewers again for their detailed assessment. Their comments prompted substantial improvements in the accuracy of the citations, methodological transparency, statistical interpretation, and alignment of the conclusions with the study design.

# Reviewer report: Reviewer response for version 2

**DOI:** 10.5256/f1000research.203333.r498510

**Published:** 2026-07-29

**Authors:** Alicia Nevriana

**Affiliations:** 1 Karolinska Institutet, Stockholm, Sweden

This study aims to determine the differences in cognitive performance between people with and without Hikikomori (prolonged social withdrawal) using data from Oman. While the theme is interesting, some areas might need clarification that the authors could consider below:

 Introduction

- Paragraph 1: ‘This condition is often comorbid with psychiatric disorders such as social anxiety disorder (SAD) and depression...’ Could the authors please clarify what ‘this condition’ refers to? Because except for the last reference (Teo, 2020), the rest of the references do not seem to study hikikomori?
- Paragraph 2: It would be great if the authors could give a reasoning on why it is important to use HiDE-I.
- Paragraph 2: ‘Additionally, the increased sensitivity to social evaluation, a hallmark of SAD, reinforces avoidant behaviors in individuals with Hikikomori (Sakamoto et al., 2005).’ I am not sure if this sentence belongs here. What is the link with HiDE-I and/or clinically classified case of hikikomori?
- Paragraph 3: ‘Research shows that social withdrawal in Hikikomori is strongly associated with psychiatric comorbidities such as depression, anxiety, and avoidant personality disorder (Bowker et al., 2019; Cai et al., 2023; Chauliac et al., 2017; Pozza et al., 2019; Teo et al., 2020).’ It seems like some of the references referred to bibliometric study and protocol for systematic reviews and not direct evidence on the association between hikikomori & psychiatric disorders. Please check.
- Paragraph 3: ‘A survey found that 54.5% of those meeting Hikikomori criteria had a psychiatric disorder, with anxiety and depression being the most common.’ It seems like the primary source of this data is this reference: Koyama A, Miyake Y, Kawakami N, et al. Lifetime prevalence, psychiatric comorbidity and demographic correlates of ”hikikomori“ in a community population in Japan. Psychiatry Res 2010; 176:69–74.8 Please check.
- Paragraph 4: ‘These domains are consistently implicated in depression, anxiety, and avoidant psychopathology, which frequently co-occur with Hikikomori.’ It would be great if the authors could give a brief introduction on the current evidence on the impairments on depression, anxiety, and avoidant psychopathology. Additionally, please give a more detailed information on the current evidence of co-occurrence of hikikomori and depression/anxiety/avoidant psychopathology, e.g., what are the prevalence, what were the study population, how was it measured, etc.
- Paragraph 6: This section probably better suits the methods. Instead, a reasoning should be provided why it is necessary to assess cognitive impairments associated with hikikomori.

Methods

- Figure 1: Perhaps the terms controls could be introduced in this figure so that the reader can connect the selection of controls in the paragraph with the figure.
- Please give a brief description of what kind of questions were asked with HiDE-I and how the scoring system was to categorise hikikomori cases.
- Has CANTAB been validated in Omani settings?
- Please give brief description of how the demographic and socioeconomic information was obtained (e.g., what were the questions, groupings, etc).
- Since the outcomes are linear, wouldn’t it have been better if the sample size was calculated based on the comparison of two means?

Discussion

- Gender-based differences: I did not see any specific analysis (e.g., interaction) to assess such differences. Could the authors please clarify from which analyses these conclusions were drawn? Additionally, given no such formal analyses were performed, perhaps the implications should focus on the actual findings of the study and not be broadened with gender differences (which this study was not aimed to do).
- Similarly with the socioeconomic factors. Given that the focus of the article is on hikikomori and cognitive performance, if there were no formal analysis on the differences in the link between hikikomori and cognitive performance stratified by socioeconomic factors, perhaps this section should be toned down.
- Limitations: is there a reason why correction for multiple testing was not performed if it becomes a concern for raising type I error?
- Limitations: please elaborate the impact of potential selection bias to the findings (e.g., will the findings be overestimated/underestimated)? Similarly, what would the impact be on the findings by not including psychiatric comorbidities and medication status?
- While the suggestion of neuroimaging in future studies might be interesting, it would be great if the authors could give justification why it would be necessary/needed?

Is the work clearly and accurately presented and does it cite the current literature?

Partly

If applicable, is the statistical analysis and its interpretation appropriate?

Partly

Are all the source data underlying the results available to ensure full reproducibility?

Partly

Is the study design appropriate and is the work technically sound?

Yes

Are the conclusions drawn adequately supported by the results?

Partly

Are sufficient details of methods and analysis provided to allow replication by others?

No

Reviewer Expertise:

psychiatric epidemiology

I confirm that I have read this submission and believe that I have an appropriate level of expertise to confirm that it is of an acceptable scientific standard, however I have significant reservations, as outlined above.

# Response

**Published:** 2026-08-26

**Authors:** Nutaila Al Kharusi

**Affiliations:** Family Medicine Public Health, Sultan Qaboos University College of Medicine and Health Science, Muscat, Muscat Governorate, Oman

We thank the reviewer for the careful and constructive assessment of our manuscript. We have addressed each comment and revised the Introduction, Methods, Results, Discussion, and Limitations accordingly. Our point-by-point responses are provided below.

 Introduction

 Comment 1

 “This condition is often comorbid with psychiatric disorders such as social anxiety disorder (SAD) and depression...” Could the authors please clarify what “this condition” refers to? Because except for the last reference (Teo, 2020), the rest of the references do not seem to study Hikikomori?

**Response:**

 Thank you for identifying this ambiguity. “This condition” referred to Hikikomori. We agree that the original sentence combined two distinct claims: psychiatric comorbidity in Hikikomori and cognitive impairment in depression and anxiety. Most of the original references supported the second claim rather than directly examining Hikikomori. We have replaced “this condition” with “Hikikomori,” separated the two claims, and assigned appropriate references to each.

 The revised text reads:

 “Hikikomori frequently co-occurs with psychiatric disorders, including mood and anxiety disorders (Koyama et al., 2010; Teo et al., 2020). Depression and anxiety are themselves associated with impairments in executive function, attention, and memory (Airaksinen et al., 2005; Bourke et al., 2012; Rock et al., 2014).”

 Comment 2

 It would be great if the authors could give a reasoning on why it is important to use HiDE-I.

**Response:**

 Thank you. We have clarified that the HQ-25 is a self-report screening measure, whereas the HiDE-I provides a structured clinician-administered assessment of withdrawal behaviour, duration, distress, and functional impairment. The HiDE-I was therefore used to determine case status independently of HQ-25 screening scores.

 The revised text reads:

 “Because the HQ-25 is a self-report screening measure intended to support rather than replace clinical assessment, case status was determined using the clinician-administered Hikikomori Diagnostic Evaluation Interview (HiDE-I). The interview assesses the pattern and duration of withdrawal and associated distress or functional impairment, permitting classification as pathological, at-risk, resembling, or non-case. In the companion diagnostic-performance study, HQ-25 sensitivity and specificity varied across thresholds and reference definitions; therefore, HiDE-I classification was used for case ascertainment in the present cognitive study.”

 Comment 3

 “Additionally, the increased sensitivity to social evaluation, a hallmark of SAD, reinforces avoidant behaviors in individuals with Hikikomori...” I am not sure if this sentence belongs here. What is the link with HiDE-I and/or clinically classified cases of Hikikomori?

**Response:**

 We agree that this sentence did not explain the HiDE-I or its role in case classification and interrupted the logical flow of the paragraph. We have therefore removed it from this location. We have also avoided relocating it as an established explanation because sensitivity to social evaluation was not directly measured in the present study.

 Comment 4

 Some references appear to be bibliometric studies or systematic-review protocols rather than direct evidence of an association between Hikikomori and psychiatric disorders.

**Response:**

 Thank you. We reviewed the cited references and agree that Cai et al. (2023) is a bibliometric publication and Pozza et al. (2019) is a systematic-review protocol; neither should be presented as primary empirical evidence of psychiatric comorbidity. We have removed these references from the relevant empirical claim and revised the wording more cautiously.

 The revised text reads:

 “Studies of Hikikomori have reported co-occurring mood, anxiety, and other psychiatric disorders, although estimates vary according to the population, case definition, and assessment method (Koyama et al., 2010; Teo et al., 2020).”

 Comment 5

 The primary source of the 54.5% statistic appears to be Koyama et al. (2010).

**Response:**

 Thank you. We verified that the 54.5% estimate originates from Koyama et al. (2010) and have cited the primary study directly. We have also clarified the study population, assessment context, and meaning of the estimate.

 The revised text reads:

 “In a Japanese community survey of adults aged 20–49 years, 54.5% of respondents with a lifetime history of Hikikomori had also experienced a lifetime mood, anxiety, impulse-control, or substance-related disorder (Koyama et al., 2010).”

 Comment 6

 Please provide more information about cognitive impairment in depression, anxiety, and avoidant psychopathology and about their co-occurrence with Hikikomori.

**Response:**

 We agree and have expanded this section with concrete information about the population, assessment method, and magnitude of the relevant findings. We have also avoided treating cognitive findings from depression and anxiety studies as direct evidence about Hikikomori.

 The revised text reads:

 “Depression is associated with measurable differences in executive function, memory, and attention. A meta-analysis of studies using CANTAB reported moderate cognitive deficits among currently depressed participants relative to controls, with Cohen’s d values ranging approximately from −0.34 to −0.65; some executive and attentional deficits also persisted during remission (Rock et al., 2014). Psychiatric comorbidity is also common in some Hikikomori populations. In a Japanese community survey using structured diagnostic assessment, 54.5% of respondents with a lifetime history of Hikikomori had experienced a lifetime psychiatric disorder (Koyama et al., 2010). These findings make emotional symptoms and psychiatric comorbidity important considerations when interpreting cognitive differences associated with Hikikomori.”

 We did not add an unverified prevalence estimate specifically for avoidant personality disorder because the available sources did not provide a sufficiently clear primary estimate for the population examined.

 Comment 7

 The description of CANTAB domains would be better placed in Methods. The Introduction should instead explain why assessing cognition in Hikikomori is necessary.

**Response:**

 We agree. We moved the detailed description of the CANTAB domains to the Methods and replaced it in the Introduction with a rationale for assessing cognition.

 The revised text reads:

 “Assessing cognition in Hikikomori is important because difficulties in planning, sustained attention, processing speed, or memory could hinder re-engagement in education, employment, and social activity. Objective assessment can also help determine whether the observed profile is selective rather than uniformly lower across cognitive domains and can inform hypotheses for longitudinal and intervention research. Because the present study is cross-sectional, it cannot determine whether cognitive differences precede or follow prolonged withdrawal.”

 Methods

 Comment 8

 Perhaps the term “controls” could be introduced in Figure 1 so that readers can connect the selection of controls in the text with the figure.

**Response:**

 Thank you. We have standardized the terminology across Figure 1 and the manuscript. Because the comparison group included psychiatric patients as well as non-patient attendees, we considered “HiDE-I-negative non-cases” more precise than “healthy controls.” Figure 1 now labels the comparison group as “HiDE-I-negative non-cases,” and the subsection previously titled “Selection of controls” has been changed to “Selection of non-cases.”

 Comment 9

 Please describe the types of questions included in the HiDE-I and the scoring or classification system used to categorize cases.

**Response:**

 We agree and have added a concise description of the interview domains and the exact three positive categories included in the study’s case definition.

 The revised text reads:

 “The HiDE-I is a structured clinician-administered interview assessing the frequency and purpose of outings, duration of withdrawal, social participation and interaction, and distress, concern by others, or functional impairment associated with withdrawal. For this neurocognitive study, participants classified as pathological, at-risk, or resembling Hikikomori were included as HiDE-I-positive cases, irrespective of HQ-25 score; participants meeting none of these categories were classified as non-cases. Pathological Hikikomori involved withdrawal lasting at least six months with significant distress or functional impairment. At-risk and resembling classifications captured emerging or subthreshold withdrawal presentations according to the administered HiDE-I criteria.”

 We use “determine” or “classify case status” rather than “confirm case status” because the study combined pathological, at-risk, and resembling presentations rather than restricting the case group to confirmed pathological cases.

 Comment 10

 Has CANTAB been validated in Omani settings?

**Response:**

 Thank you. Omani population-specific normative data and formal local validation were not available for the present study. We have stated this directly and added it as a limitation. CANTAB was selected because of its computerized, largely non-verbal administration and standardized response recording; however, these features do not eliminate cultural, educational, or contextual influences.

 The revised limitation reads:

 “Omani population-specific normative data and formal local validation for this CANTAB battery were not available to this study. Although the computerized, largely non-verbal format reduces some language and examiner effects, it does not eliminate cultural, educational, or contextual influences. Local psychometric and normative studies are therefore needed.”

 Comment 11

 Please describe how demographic and socioeconomic information was obtained, including the questions and groupings.

**Response:**

 We agree and have specified the mode of completion, variables collected, and analytical categories. The Methods and Table 2 have been aligned so that the reported groupings are consistent.

 The revised text reads:

 “Participants completed a structured self-report sociodemographic questionnaire covering age, gender, marital status, education, monthly household income, employment status, governorate of residence, and whether they had children. For the present analysis, age was grouped as 18–29, 30–39, and ≥40 years; income as <1,000, 1,000–1,999, 2,000–2,999, and ≥3,000 OMR; employment as employed or unemployed; marital status as never married or ever married; and education as <12, 12, or >12 years. Governorates represented in the sample are listed in Table 2.”

 Comment 12

 Since the outcomes are linear, would it not have been better to calculate the sample size based on a comparison of two means?

**Response:**

 We agree. The odds-ratio-based Episheet calculation was developed for the broader parent case–control study and was not tailored to the continuous CANTAB outcomes examined in this neurocognitive sub-study. We have clarified this distinction and removed post-hoc power statements based on observed effect sizes.

 The revised text reads:

 “Sample size for the broader parent case–control study was calculated a priori using Rothman’s Episheet and was based on odds-ratio detection, assuming a control-to-case ratio of 1:1, a Zα value of 1.96, an exposure prevalence of 0.15, and 80% power to detect an odds ratio of 2.0. This calculation was not specifically tailored to the continuous CANTAB outcomes examined in the present neurocognitive sub-study. A two-group calculation based on an anticipated mean difference and variance would have been more directly appropriate.

 The achieved case-to-non-case ratio was 54:96 (approximately 1:1.8), rather than the 1:1 ratio assumed in the parent-study calculation. With a fixed total sample size and similar group variances, unequal allocation generally provides less precision than equal allocation; however, recruiting additional non-cases can improve precision when the number of available cases is constrained. Because the original calculation was not based on the continuous CANTAB outcomes, the power implications of the achieved ratio were not quantified. The study may therefore have had limited precision for detecting small effects, and non-significant findings should not be interpreted as evidence of equivalence.”

 Discussion

 Comment 13

 No formal analysis was performed to assess gender-based differences, and the conclusion that social withdrawal may affect both genders similarly is not supported.

**Response:**

 We agree. Gender was included as a covariate, but no case-status-by-gender interaction or gender-stratified analysis was performed. We have removed the conclusion that social withdrawal affects both genders similarly and limited the interpretation to the analysis performed.

 The revised text reads:

 “Gender was included as a covariate in the adjusted regression models, but no case-status-by-gender interaction or gender-stratified analysis was performed. The present results therefore cannot determine whether the association between HiDE-I case status and cognitive performance differs by gender. Future studies with larger samples should formally examine gender as a potential moderator of this association.”

 Comment 14

 The socioeconomic discussion should be toned down because no socioeconomic-stratified analysis was performed.

**Response:**

 We agree. Income and employment were examined descriptively and included as covariates, but no interaction, mediation, or stratified analysis tested socioeconomic causation or effect modification. We have removed the causal interpretation.

 The revised text reads:

 “Cases and non-cases differed descriptively in their employment and household-income distributions (Table 2). Employment and income were included as covariates in the adjusted cognitive models; however, no interaction, mediation, or socioeconomic-stratified analysis was performed. The present study therefore cannot determine whether socioeconomic factors caused social withdrawal or modified the association between HiDE-I case status and cognitive performance. These findings should be interpreted as characteristics of the study sample rather than evidence of a causal socioeconomic mechanism.”

 Comment 15

 Why was correction for multiple testing not performed if type I error was a concern?

**Response:**

 Thank you. No multiplicity correction was reported in the previous version. We have now applied multiplicity sensitivity analyses to the 11 adjusted case-status tests. Using the Bonferroni family-wise threshold of α=.0045, visual-processing accuracy, planning efficiency, response latency, and delayed recall remained statistically significant. Immediate recall, with an unadjusted p=.019, did not survive the Bonferroni correction and is now interpreted as suggestive rather than confirmatory. We also conducted a Benjamini–Hochberg false-discovery-rate sensitivity analysis.

 The Statistical Analysis section now states:

 “Because 11 cognitive outcomes were examined, multiplicity was assessed using a Bonferroni family-wise significance threshold of α=.0045 (.05/11). A Benjamini–Hochberg false-discovery-rate correction (q=.05) was also conducted as a sensitivity analysis.”

 The Results now state:

 “After Bonferroni correction, the associations with visual-processing accuracy (p=.001), planning efficiency (p=.001), response latency (p=.001), and delayed recall (p=.003) remained statistically significant. Immediate recall (unadjusted p=.019) did not meet the Bonferroni-adjusted threshold and should therefore be interpreted as suggestive.”

 Comment 16

 Please elaborate on the likely impact of selection bias and the failure to include psychiatric comorbidity and medication status.

**Response:**

 We agree and have expanded the Limitations. Recruitment from a tertiary behavioral-medicine clinic may overrepresent help-seeking or clinically complex participants, potentially enlarging the observed cognitive differences relative to a community sample. Conversely, psychiatric morbidity among non-case patients could attenuate case–non-case differences. Consequently, the net direction of selection bias cannot be determined with certainty.

 All 150 CANTAB participants completed the DASS-21, allowing an exploratory post-hoc analysis of current depression, anxiety, and stress symptoms. However, DASS-21 is a symptom measure rather than a structured diagnostic interview and does not control diagnosis type, neurodevelopmental conditions, substance use, or medication exposure. We have therefore avoided claiming that psychiatric confounding was eliminated.

 The revised limitation reads:

 “Recruitment from one tertiary behavioral-medicine clinic may overrepresent help-seeking or clinically complex participants and limits generalizability to community Hikikomori. The direction of selection bias in the cognitive comparisons is uncertain: greater clinical severity among cases could enlarge the observed differences, whereas psychiatric morbidity among non-case patients could attenuate them. Formal psychiatric diagnoses and medication exposure were not systematically incorporated into the primary cognitive models. Depression, anxiety, neurodevelopmental conditions, other psychiatric disorders, and psychotropic medication may affect attention, processing speed, and memory; imbalance between groups could therefore confound the magnitude or attribution of the observed differences. DASS-21 symptom data permitted a limited exploratory assessment of current emotional distress but did not substitute for structured diagnoses or medication histories.”

 Comment 17

 Please justify why neuroimaging would be necessary or useful in future studies.

**Response:**

 Thank you. We agree that the previous recommendation for neuroimaging was insufficiently justified. We have clarified that neuroimaging could complement behavioral testing by examining whether differences in attention, planning, and memory correspond to differences in the neural systems supporting these functions. We also emphasize that neuroimaging should be combined with longitudinal assessment and structured measurement of psychiatric diagnoses, neurodevelopmental conditions, emotional symptoms, and medication exposure. Cross-sectional imaging alone would not establish causality or a Hikikomori-specific mechanism.

 The revised text reads:

 “Future research should combine longitudinal cognitive assessment with structured measurement of psychiatric diagnoses, neurodevelopmental conditions, emotional symptoms, and medication exposure. Structural and functional neuroimaging may help determine whether the observed behavioral differences correspond to changes in neural systems supporting attention, planning, and memory (Zovetti et al., 2021). Longitudinal designs would be required to examine whether these cognitive and neural differences precede prolonged withdrawal, emerge following withdrawal, or change during recovery or intervention. Cross-sectional neuroimaging alone would not establish a Hikikomori-specific mechanism. Because exposure and outcome were measured cross-sectionally, the present study cannot determine whether cognitive differences preceded withdrawal, followed it, or arose from shared psychiatric, developmental, medication-related, or social factors.”

 We again thank the reviewer for these detailed recommendations. They helped us improve the accuracy of the citations, methodological transparency, statistical interpretation, and alignment of the Discussion with the analyses conducted.
